# A syndemic approach to assess the effect of substance use and social disparities on the evolution of HIV/HCV infections in British Columbia

**DOI:** 10.1371/journal.pone.0183609

**Published:** 2017-08-22

**Authors:** Zahid Ahmad Butt, Nabin Shrestha, Stanley Wong, Margot Kuo, Dionne Gesink, Mark Gilbert, Jason Wong, Amanda Yu, Maria Alvarez, Hasina Samji, Jane A. Buxton, James C. Johnston, Victoria J. Cook, David Roth, Theodora Consolacion, Michelle Murti, Travis S. Hottes, Gina Ogilvie, Robert Balshaw, Mark W. Tyndall, Mel Krajden, Naveed Z. Janjua, for the BC Hepatitis Testers Cohort

**Affiliations:** 1 School of Population and Public Health, University of British Columbia, Vancouver, British Columbia, Canada; 2 British Columbia Centre for Disease Control, Vancouver, British Columbia, Canada; 3 Dalla Lana School of Public Health, University of Toronto, Toronto, Ontario, Canada; 4 BCCDC Public Health Laboratory, Vancouver, British Columbia, Canada; 5 Fraser Health, Surrey, British Columbia, Canada; Yale University Yale School of Public Health, UNITED STATES

## Abstract

**Background:**

Co-occurrence of social conditions and infections may affect HIV/HCV disease risk and progression. We examined the changes in relationship of these social conditions and infections on HIV and hepatitis C virus (HCV) infections over time in British Columbia during 1990–2013.

**Methods:**

The BC Hepatitis Testers Cohort (BC-HTC) includes ~1.5 million individuals tested for HIV or HCV, or reported as a case of HCV, HIV, HBV, or tuberculosis linked to administrative healthcare databases. We classified HCV and HIV infection status into five combinations: HIV-/HCV-, HIV+monoinfected, HIV-/HCV+seroconverters, HIV-/HCV+prevalent, and HIV+/HCV+.

**Results:**

Of 1.37 million eligible individuals, 4.1% were HIV-/HCV+prevalent, 0.5% HIV+monoinfected, 0.3% HIV+/HCV+ co-infected and 0.5% HIV-/HCV+seroconverters. Overall, HIV+monoinfected individuals lived in urban areas (92%), had low injection drug use (IDU) (4%), problematic alcohol use (4%) and were materially more privileged than other groups. HIV+/HCV+ co-infected and HIV-/HCV+seroconverters were materially most deprived (37%, 32%), had higher IDU (28%, 49%), problematic alcohol use (14%, 17%) and major mental illnesses (12%, 21%). IDU, opioid substitution therapy, and material deprivation increased in HIV-/HCV+seroconverters over time. In multivariable multinomial regression models, over time, the odds of IDU declined among HIV-/HCV+prevalent and HIV+monoinfected individuals but not in HIV-/HCV+seroconverters. Declines in odds of problematic alcohol use were observed in HIV-/HCV+seroconverters and coinfected individuals over time.

**Conclusions:**

These results highlight need for designing prevention, care and support services for HIV and HCV infected populations based on the evolving syndemics of infections and social conditions which vary across groups.

## Introduction

In Canada, an estimated 230,000–450,000(0.66%–1.3%) individuals were infected with hepatitis C virus (HCV)[1] and about 75,500 were living with HIV at the end of 2014 [2]. Both HCV and HIV are associated with significant morbidity[3] and mortality [4, 5]. HIV and HCV coinfected individuals have an increased risk of cirrhosis and decompensated liver disease [3], cardiovascular disease [6], Chronic Kidney Disease [7], and osteoporotic fractures[8]. In 2015, an estimated 400,000 people died from HCV globally [9], whereas HIV was responsible for 1.1 million deaths [10]. Recent data from North America shows that HCV related mortality has surpassed HIV-related mortality [11]. New highly effective and well tolerated directly acting antivirals (DAA) are expected to reduce the HCV related morbidity and mortality with optimal scale up of treatment and screening to identify undiagnosed individuals living with HCV [12].

In the developed world, HCV and HIV infections share common transmission routes and predisposing vulnerabilities; therefore, HCV-HIV co-infection is relatively high [13]. Poor social conditions, vulnerabilities and/or other infections (e.g. addictions, mental illness, HBV, TB) are common in those with HCV and/or HIV [14, 15]. Integration of services related to HCV, HIV, substance use, and mental illness could improve health outcomes and reduce onward transmission of both infections[16]. However, the distribution of social conditions and infections is not uniform across all HCV and HIV population groups [17]. Furthermore, the distribution of these co-occurring and predisposing conditions has not been described at a broader population level, mainly due to lack of population level data. Such data could inform the delivery of integrated services for various groups of people according to their specific needs based on multiple co-occurring conditions and infections. In addition, infections and social conditions continue to evolve and there is limited data to describe and understand such evolution at the population level. In British Columbia, we have assembled a population-base cohort on HCV and HIV testing and diagnoses since the early 1990s to address the knowledge gaps highlighted above.

Using this cohort, we describe the co-occurrence of HCV and HIV infections, related social conditions and disparities in British Columbia. We also assessed trends in the distribution of various social conditions, disparities and co-infections by HCV and HIV infection status between 1990 and 2013.

## Materials and methods

The British Columbia Hepatitis Testers Cohort (BC-HTC) includes all individuals tested for HCV or HIV at the BCCDC Public Health Laboratory (BCCDC-PHL) or reported to public health as a confirmed case of HCV, Hepatitis B Virus (HBV), HIV/AIDS or active TB (Tuberculosis). This cohort is linked with provincial healthcare administrative databases including medical visits, hospitalizations, prescription drugs, cancers and deaths (S1 Table). Almost all HIV and HCV testing in BC is performed at the BCCDC-PHL. All dispensed prescriptions in BC are recorded in a central system called PharmaNet, regardless of the payer. Detailed characteristics of the cohort and related data are presented elsewhere [17, 18].

### Eligibility criteria

Individuals with at least one HCV (antibody, HCV RNA, or genotype) or HIV test between 1992–2013, or those reported as an HCV (1990–2013) or HIV (1980–2013) case to public health were included in this analysis.

### Definitions

An individual testing positive for HCV antibodies, HCV RNA or genotype or who was reported as an HCV case to public health was considered as HCV case [18]. Participants who were HCV antibody positive at their first test on record were considered to have prevalent infection while individuals with a negative test followed by a positive test were considered seroconverters [17]. An individual included in the Provincial HIV/AIDS Information System, or who had a positive HIV lab test result was considered a case based on provincial HIV laboratory test interpretation guidelines [18]. Additional HIV cases were ascertained through a validated algorithm requiring two medical visits or an hospitalization [18]. HBV and active TB diagnoses were based on provincial and national guidelines [18]. Socioeconomic status was assessed using the Québec Index of Material and Social Deprivation which is based on Canadian Census Data on small area units [19]. The material component consisted of indicators for education, employment and income whereas the social component consisted of indicators related to marital status and family structure. Assessment of injection drug use (IDU), major mental illness, and problematic alcohol use was based on diagnostic codes (S2 Table) for medical visits and hospitalizations in respective databases. Opioid Substitution Therapy (OST) was based on prescriptions recorded in PharmaNet. IDU, problematic alcohol use, OST, and mental illnesses were evaluated across all data prior (ever/baseline) to or within 3 years (recent) of the diagnosis date or last negative test.

### Statistical analysis

HIV and HCV testers were classified into: tested negative for HCV and HIV, tested negative for HCV but not tested for HIV and tested for HIV but not tested for HCV (HIV-/HCV), HIV mono-infected (HIV+/HCV-), prevalent HCV (HIV-/HCV+ prevalent), HCV seroconverters (HIV-/HCV+ seroconverters), and HIV-HCV co-infected (HIV+/HCV+ coinfected). We described the distribution of characteristics of HCV, HIV, and coinfected groups overall and by time periods for stratified analyses (<2000, 2000–2004, 2005–2009 and 2010–2013). We choose these time periods to reflect changes in HIV treatment strategies and HCV testing patterns in British Columbia over time [17, 20].

We constructed multivariable multinomial regression models measuring associations of various factors with four positive categories for HIV and HCV infections (HIV+ monoinfected, HIV+/HCV+ coinfected, HIV-/HCV+ seroconverters and HIV-/HCV+ prevalent) compared to the one negative category (HIV-/HCV-). As the one negative category (HIV-/HCV-) also consisted of those individuals that were tested for either HIV and HCV and not tested for one of these infections, and could lead to misclassification, we ran a sensitivity analysis excluding these testers (S3 Table). However, the analysis yielded similar results as our main analysis. Characteristics of participants that were evaluated in various models included gender/sex, birth year, age at diagnosis, urbanicity, active TB, HBV, IDU, OST, problematic alcohol use, mental illness, depression, psychosis and material and social deprivation. Separate multivariable models were constructed for recent history (within 3 years) and baseline (past history) values of these characteristics with HIV and HCV categories as outcomes. For interpretation, adjusted Odds Ratios (aORs) and their respective 95% Confidence Intervals (CIs) were estimated. We performed an additional cross sectional stratified analysis by year of HCV/HIV diagnosis to examine shifts in factor epidemiology and to account for changes in HCV and HIV co-infection status overtime. Four different multivariate models were generated to examine the trends across these time periods. Analyses were performed in SAS/STAT software version [9.4] and R.

Study was approved by Behavioral Research Ethics Board (H15-01776) at the University of British Columbia.

## Results

The analysis included 1,376,989 individuals. Of these, 1,302,877 (94.6%) were either negative for both HCV and HIV or not tested for HCV or HIV, 7013 (0.5%) were HIV-/HCV+ seroconverters, 56,074 (4.1%) were HIV-/HCV+prevalent individuals, 4639 (0.3%) were positive for both HCV and HIV, and 6386 (0.5%) were positive for HIV only.

### Distribution of characteristics across HIV and HCV groups

Males were more common in all HIV and HCV positive groups and less common among negatives. The majority of HIV-/HCV+ seroconverters (71%) and HIV-/HCV- individuals (60%) were younger and born after 1964, whereas most of HIV+/HCV+ co-infected (57%), HIV-/HCV+ prevalent (66%) and HIV+ monoinfected (48%) were older and born during 1945–1964. More than 90% of HIV+ monoinfected and HIV+/HCV+ individuals lived in urban areas, while this proportion was 84% for HIV-/HCV+ prevalent and 86% for HIV-/HCV+ seroconverters. A higher proportion of HIV-/HCV+ seroconverters (32.3%), HIV-/HCV+ prevalent (27.5%) and HIV+/HCV+ (37%), were materially most deprived (in the 5^th^ quintile) while a higher percentage of HIV+ monoinfected individuals were materially most privileged (33% in 1^st^ quintile). HIV+ monoinfected individuals had less IDU (4%) than HIV-/HCV+ seroconverters (49%), HIV+/HCV+ (28%) and HIV-/HCV+ prevalent individuals (13.7%). A higher proportion of major mental health illnesses were observed in HIV-/HCV+ seroconverters (21%) and HIV+/HCV+ (12%) individuals compared to other groups (5.7–8.1%) (Table 1). A similar trend was observed for these factors at baseline (S4 Table).

**Table 1:** Characteristics of participants by HIV and HCV status in BC hepatitis testers cohort.

	HIV+/HCV+	HIV+/HCV-	HIV-/HCV+ seroconverters	HIV-/HCV+ prevalent	HIV-/HCV-
Variable	N (%)	N (%)	N (%)	N (%)	N (%)
(row percent)	4639(0.3)	6386(0.5)	7013(0.5)	56074(4.1)	1302877(94.6)
**Sex**					
Female	1352(29.1)	956(15)	3120(44.5)	19086(34)	765346(58.7)
Male	3286(70.8)	5430(85)	3893(55.5)	36983(66)	537363(41.2)
Unknown	1(0)	0(0)	0(0)	5(0)	168(0)
**Birth year**					
< 1945	106(2.3)	474(7.4)	123(1.8)	6137(10.9)	165710(12.7)
1945–1964	2635(56.8)	3058(47.9)	1901(27.1)	36762(65.6)	356946(27.4)
> 1964	1898(40.9)	2854(44.7)	4989(71.1)	13175(23.5)	780221(59.9)
**Age at diagnosis**					
<15	13(0.3)	97(1.5)	17(0.2)	425(0.8)	20573(1.6)
15–24	580(12.5)	460(7.2)	1306(18.6)	2298(4.1)	174233(13.4)
25–34	1702(36.7)	1911(29.9)	2686(38.3)	9794(17.5)	364607(28)
35–44	1688(36.4)	2089(32.7)	1888(26.9)	18076(32.2)	280038(21.5)
45–54	552(11.9)	1178(18.4)	810(11.5)	15804(28.2)	190520(14.6)
>54	104(2.2)	651(10.2)	306(4.4)	9677(17.3)	272906(20.9)
**Urban**					
Unknown	87(1.9)	172(2.7)	162(2.3)	2525(4.5)	29015(2.2)
No	289(6.2)	334(5.2)	785(11.2)	6590(11.8)	131070(10.1)
Yes	4263(91.9)	5880(92.1)	6066(86.5)	46959(83.7)	1142792(87.7)
**Social deprivation quintile at time of** **test**					
Unknown	150(3.2)	202(3.2)	178(2.5)	2396(4.3)	21030(1.6)
Q1 (most privileged)	259(5.6)	538(8.4)	592(8.4)	6111(10.9)	235246(18.1)
Q2	405(8.7)	656(10.3)	675(9.6)	7294(13)	229929(17.6)
Q3	584(12.6)	717(11.2)	1024(14.6)	9391(16.7)	234744(18)
Q4	930(20)	1205(18.9)	1409(20.1)	11626(20.7)	269127(20.7)
Q5 (most deprived)	2311(49.8)	3068(48)	3135(44.7)	19256(34.3)	312801(24)
**Material deprivation quintile at time of test**					
Unknown	150(3.2)	202(3.2)	178(2.5)	2396(4.3)	21030(1.6)
Q1 (most privileged)	659(14.2)	2101(32.9)	826(11.8)	7174(12.8)	282648(21.7)
Q2	624(13.5)	1075(16.8)	970(13.8)	8769(15.6)	248346(19.1)
Q3	606(13.1)	849(13.3)	1112(15.9)	10022(17.9)	254554(19.5)
Q4	891(19.2)	938(14.7)	1665(23.7)	12303(21.9)	260362(20)
Q5 (most deprived)	1709(36.8)	1221(19.1)	2262(32.3)	15410(27.5)	235937(18.1)
**Illicit Drug Use^a^**					
No	3044(65.6)	6070(95.1)	3272(46.7)	47015(83.8)	1272255(97.6)
Yes	1595(34.4)	316(4.9)	3741(53.3)	9059(16.2)	30622(2.4)
**IDU^a^**					
No	3242(69.8)	6109(95.7)	3564(50.8)	48380(86.3)	1276106(97.9)
Yes	1397(28.1)	277(4.3)	3449(49.2)	7694(13.7)	26771(2.1)
**OST^a^**					
No	4238(91.4)	6355(99.5)	5253(74.9)	52897(94.3)	1294249(99.3)
Yes	401(8.6)	31(0.5)	1760(25.1)	3177(5.7)	8628(0.7)
**Major mental illness^a^**					
No	4070(87.7)	5866(91.9)	5512(78.6)	51569(92)	1228554(94.3)
Yes	569(12.3)	520(8.1)	1501(21.4)	4505(8)	74323(5.7)
**Depression^a^**					
No	3590(77.4)	5283(82.7)	4262(60.8)	44794(79.9)	1091992(83.8)
Yes	1049(22.6)	1103(17.3)	2751(39.2)	11280(20.1)	210885(16.2)
**Psychosis^a^**					
No	4447(95.9)	6264(98.1)	6565(93.6)	54818(97.8)	1285280(98.6)
Yes	192(4.1)	122(1.9)	448(6.4)	1256(2.2)	17597(1.4)
**Problematic alcohol use^a^**					
No	3962(85.4)	6148(96.3)	5841(83.3)	51381(91.6)	1277040(98)
Yes	677(14.6)	238(3.7)	1172(16.7)	4693(8.4)	25837(2)
**Active TB^a^**					
No	4629(99.8)	6370(99.7)	7007(99.9)	56016(99.9)	1300778(99.8)
Yes	10(0.2)	16(0.3)	6(0.1)	58(0.1)	2099(0.2)
**Hepatitis B^a^**					
No	4587(98.9)	6319(99)	6921(98.7)	55673(99.3)	1296688(99.5)
Yes	52(1.1)	67(1)	92(1.3)	401(0.7)	6189(0.5)

Abbreviations: IDU, injection drug use; OST, opioid substitution therapy.

^a^ Factor assessed for past 3 years before diagnosis or last negative test.

### Distribution of characteristics by HIV and HCV groups over time

**Fig 1:**
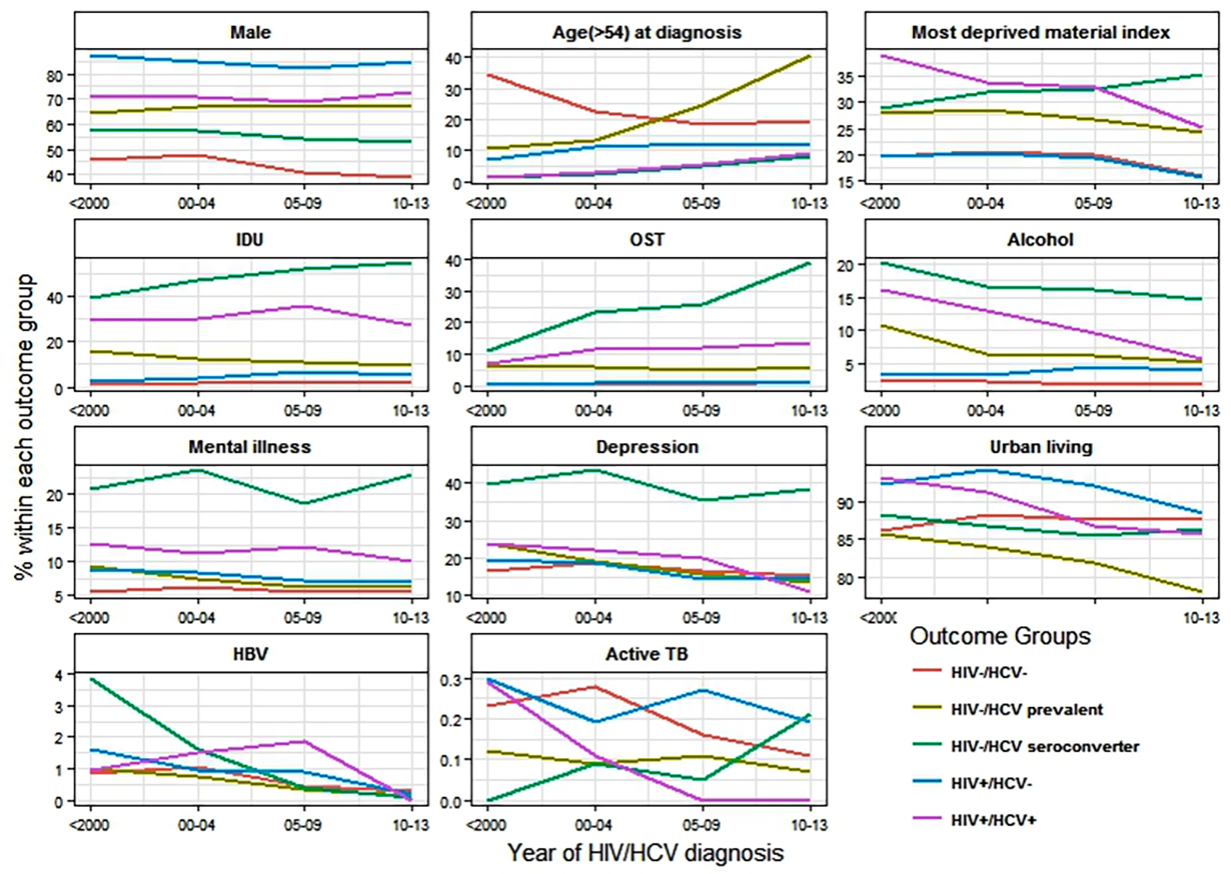
Distribution of selected factors by HIV and HCV status over time in the BC Hepatitis Testers Cohort, British Columbia, Canada, 1990–2013.

Fig 1 and S5 Table present trends of substance use, co-morbidities, mental illness and disparities among all HIV and HCV groups over time. From 1990 to 2013, Illicit drug use and IDU remained persistently high (> 40%) in HIV-/HCV+ seroconverters while this declined after 2009 for all other groups. OST increased markedly from 2000 onwards among HIV-/HCV+ seroconverters (11 to 39%) while a gradual increase was observed for HIV+/HCV+ and HIV-/HCV+ prevalent individuals. Problematic alcohol use declined among HIV+/HCV+, HIV-/HCV+seroconverters and HIV-/HCV+ prevalent individuals but increased slightly for HIV+ monoinfected individuals. Among HIV-/HCV+ seroconverters, major mental illnesses, depression and psychosis remained high while there was slight decline in other HIV/HCV groups. A decline in HBV infection was observed in all groups over time. Active TB proportion was low and showed a decreasing trend in HIV-/HCV- and HIV+/HCV+ while it increased in HIV-/HCV+ seroconverters and remained high in HIV+ monoinfected. Material deprivation also increased in HIV-/HCV+ seroconverters but declined in other groups. A slight decrease in urban residence was observed after 2010 for HIV+ monoinfected, HIV-/HCV+ prevalent and HIV+/HCV+ individuals.

### Factors associated with HIV/HCV infection groups

We developed two multivariable models which spanned the entire study period; one representing the association between baseline (past history) of a factor with HIV and HCV infection groups. The other model represented the association between recent history (within 3 years) of a factor with HIV and HCV infection groups.

In the model with recent history of risk factors, male sex, age at diagnosis, IDU, problematic alcohol use, major mental illnesses, HBV, material and social deprivation were associated with either all or one of the HIV/HCV groups compared to HIV-/HCV- (Table 2). Being male, living in an urban area and having major mental illness had the strongest association with HIV+ monoinfected individuals, while problematic alcohol use was not associated with being HIV+. Urban residence, problematic alcohol use, IDU, social and material deprivation were major predictors of HIV+/HCV+ coinfection. For HIV-/HCV+ seroconverters, magnitude of association for IDU and HBV was highest whereas problematic alcohol use was similar to HIV+/HCV+ co-infected individuals. Younger age was associated with being a HIV-/HCV+ seroconverter and a HIV+/HCV+ coinfected individual. IDU was associated with all HIV/HCV groups though magnitude of association was highest for HIV-/HCV+ seroconverters (OR = 33, 95% CI = 31.2, 35.1) followed by HIV+/HCV+ coinfected (OR = 14.7, 95% CI = 13.67, 15.97 and lowest for HIV+ monoinfected (OR = 1.6, 95% CI = 1.45, 1.89). Social deprivation was associated with all HIV/HCV groups with increasing ORs across all quintiles compared to the most privileged quintile. HIV+/HCV+ co-infected individuals, HIV-/HCV+ seroconverters, and HIV-/HCV+ prevalent showed trends of increasing ORs with increase in material deprivation. While HIV+ monoinfected had lower odds for all material deprivation quintiles, indicating that this group was least likely to be materially deprived (Table 2). The model with characteristics measured at the baseline (past history) showed similar results (data not shown), though the magnitude of ORs was higher in the model with recent history of factors.

**Table 2:** Multivariate multinomial logistic regression model for factors associated with HIV and HCV infection status in the BC Hepatitis Testers Cohort^a^.

Variable	HIV+/HCV+	HIV+ / HCV-	HIV- / HCV+ prevalent	HIV- /HCV+ seroconverters
	OR (95% CI)	OR (95% CI)	OR (95% CI)	OR (95% CI)
**Sex**				
Female	1	1	1	1
Male	2.9(2.68, 3.06)	7.4(6.96, 8.02)	2.3(2.26, 2.35)	1.4(1.37, 1.52)
**Age at diagnosis**				
<15	1.1(0.58, 2.03)	1.6(1.25, 1.94)	0.4(0.38, 0.47)	0.7(0.41, 1.14)
15–24	14.0(11.26, 17.39)	1.6(1.46, 1.88)	0.5(0.49, 0.54)	5.2(4.55, 5.91)
25–34	16.8(13.7, 20.7)	3.2(2.98, 3.58)	1.0(1.0, 1.07)	5.2(4.59, 5.87)
35–44	15.9(13.02, 19.63)	3.6(3.34, 4.0)	1.94(1.88, 1.99)	4.2(3.69, 4.74)
45–54	8.86(7.13, 11.0)	2.9(2.63, 3.20)	2.5(2.49, 2.64)	2.8(2.46, 3.24)
>54	1	1	1	1
**Urban**				
No	1	1	1	1
Yes	1.7(1.49, 1.91)	1.8(1.60, 2.00)	0.9(0.88, 0.93)	1.0(0.93, 1.09)
**IDU^b^**				
No	1	1	1	1
Yes	14.7(13.67, 15.97)	1.6(1.45, 1.89)	7.7(7.49, 8.03)	33.1(31.2, 35.1)
**Problematic alcohol use^b^**				
No	1	1	1	1
Yes	2.3(2.12, 2.59)	1.0(0.90, 1.19)	1.8(1.74, 1.88)	1.9(1.80, 2.10)
**Major mental illness^b^**				
No	1	1	1	1
Yes	0.7(0.59, 0.73)	1.2(1.14, 1.38)	0.7(0.68, 0.73)	0.9(0.82, 0.94)
**Active Tb^b^**				
No	1	1	1	1
Yes	0.6(0.30, 1.21)	1.3(0.82, 2.25)	0.4(0.30, 0.53)	0.4(0.18, 0.94)
**Hepatitis B^b^**				
No	1	1	1	1
Yes	0.9(0.71, 1.26)	1.2(0.95, 1.58)	0.8(0.74, 0.93)	2(1.61, 2.53)
**Year of diagnosis**				
>2009	1	1	1	1
2005–2009	5.6(4.61, 6.85)	2.4(2.29, 2.70)	2.8(2.74, 2.94)	3.0(2.81, 3.23)
2000–2004	26.5(22.12, 31.97)	5.1(4.78, 5.62)	7.5(7.35, 7.85)	6.5(6.10, 7.04)
<2000	138.5(116.1, 165.2)	11.6(10.82, 12.61)	22.6(21.99, 23.39)	5.5(5.14, 6.05)
**Social deprivation at time of test**				
Q1 (most privileged)	1	1	1	1
Q2	1.6(1.33, 1.83)	1.2(1.09, 1.37)	1.2(1.12, 1.21)	1.2(1.06, 1.33)
Q3	2.1(1.8, 2.40)	1.3(1.19, 1.49)	1.4(1.34, 1.44)	1.6(1.47, 1.81)
Q4	2.9(2.56, 3.39)	2.0(1.81, 2.22)	1.56(1.51, 1.61)	1.86(1.68, 2.06)
Q5 (most deprived)	5.1(4.46, 5.80)	3.7(3.33, 4.02)	2.1(2.01, 2.14)	3.0(2.74, 3.29)
**Material deprivation at time of test**				
Q1 (most privileged)	1	1	1	1
Q2	1(0.94, 1.17)	0.7(0.6, 0.7)	1.3(1.29, 1.38)	1.3(1.21, 1.46)
Q3	1(0.88, 1.11)	0.5(0.47, 0.56)	1.5(1.42, 1.52)	1.4(1.26, 1.51)
Q4	1.3(1.2, 1.46)	0.5(0.48, 0.56)	1.7(1.64, 1.74)	1.8(1.64, 1.94)
Q5 (most deprived)	2.3(2.09, 2.52)	0.7(0.62, 0.71)	2.1(2.06, 2.2)	2.3(2.13, 2.52)

Abbreviations: IDU, injection drug use.

^a^ Reference group: HIV-/HCV-.

^b^ Factor assessed for past 3 years before diagnosis or last negative test.

Additional models presented in supplementary material include: birth year in the recent model (S6 Table), age at diagnosis, depression and psychosis in the recent model (S7 Table) and age at diagnosis in the baseline model (S8 Table).

### Factors associated with HIV/HCV infection groups across time periods

**Fig 2:**
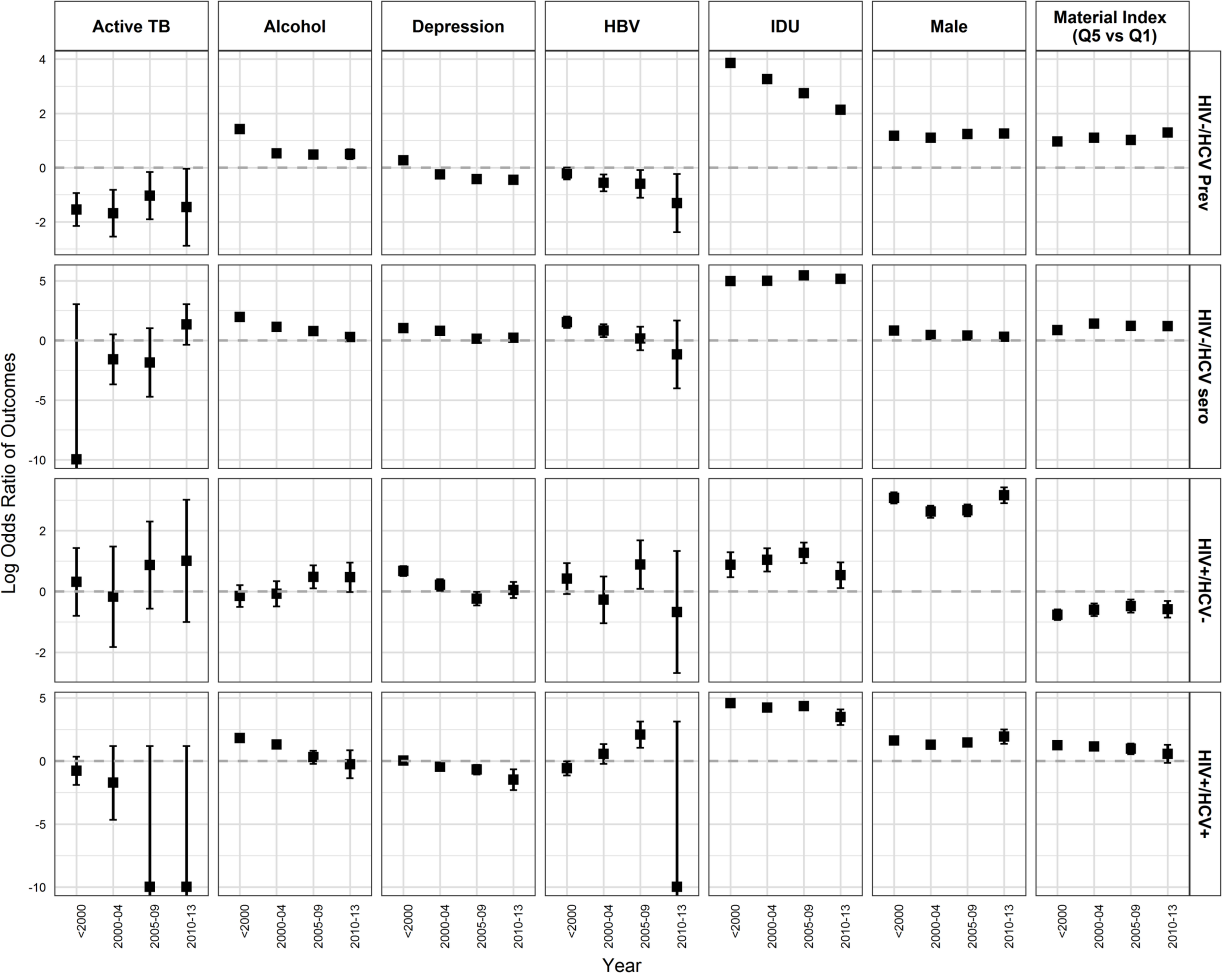
Adjusted ORs from multivariable models for factors associated with HIV and HCV infection status over time. (ORs are calculated as logarithmic function of 2). Abbreviations: HIV-/HCV Prev, HIV-/HCV+ prevalent; HIV-/HCV sero, HIV-HIV+seroconverter; HIV+/HCV-, HIV+ monoinfected.

**Fig 3:**
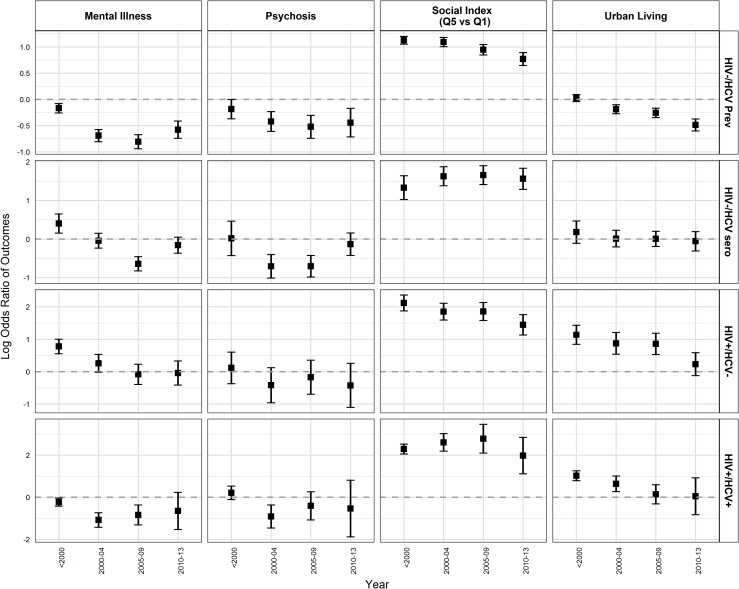
Adjusted ORs from multivariable models for factors associated with HIV and HCV infection status over time. **(**ORs are calculated as logarithmic function of 2). Abbreviations: HIV-/HCV Prev, HIV-/HCV+prevalent; HIV-/HCV sero, HIV-HIV+seroconverter; HIV+/HCV-, HIV+monoinfected.

We also developed additional multivariable models examining the relationship between characteristics of participants and HIV/HCV infection groups to assess changes over time (from before 2000 to 2013). Most of the associations persisted (S9 Table) and were similar to the overall analysis (Table 2); however, there were some notable changes. HIV-/HCV+ prevalent group were no longer associated with residence in urban areas after 2005. No association was observed between urban residence and HIV+ monoinfected and HIV+/HCV+ coinfected individuals after 2009. The magnitude of association of IDU with HIV-/HCV+ prevalent and HIV+/HCV+ coinfected individuals declined over time (Fig 2/S9 Table). The odds of problematic alcohol use declined more steeply over time for HIV-/HCV+ seroconverters and HIV+/HCV+ co-infected whereas there was no change in association with HIV-/HCV+ prevalent from 2000 onwards. HIV-/HCV+ prevalent were more likely to be born in the birth cohort of 1945–64 compared to >1964 birth cohort (S9 Table). The odds of being diagnosed (for HCV/HIV) at age 25–34 years compared to >54 years declined for HIV-/HCV+ prevalent individuals, HIV-/HCV+ seroconverters, and HIV+/HCV+ coinfected individuals with some decline in odds for HIV+ monoinfected as well (S9 Table). Active TB was significantly associated with HIV+ monoinfected and HIV-/HCV+ seroconverters from 2009 onwards (Fig 2). The association of material and social deprivation with HIV/HCV infection groups (Fig 2 and Fig 3) remained the same in these models as compared to the overall analysis.

## Discussion

This study characterizes the co-occurrence of and trends in HIV and HCV infection, substance use, mental illness and associated underlying socioeconomic disparities at the population level in British Columbia. Overall, HIV+ monoinfected individuals were predominantly male urban residents, had lower substance use, and were socially deprived but materially more privileged, while HIV-/HCV+ seroconverters and HIV+/HCV+ coinfected had higher substance use, mental illness, and were materially more deprived. HIV-/HCV+ prevalent group were older and had relatively lower substance use. Tailored integrated care packages and targeted interventions can be developed keeping in mind the different risk profiles of each group.

Overall, HIV+ monoinfected individuals were primarily male, living in urban areas, and were more likely to be socially but not materially deprived [15, 21, 22]. Urban residence and migration to urban areas by HIV infected individuals predominantly to Vancouver city in British Columbia could be due to real or perceived accessibility to HIV related health care services [23]. In our cohort, the HIV+ monoinfected population are most likely individuals who are MSM based on the higher proportion of males and a sub analysis of HIV data. Social deprivation among HIV+ monoinfected may indicate stigma related to HIV or sexual orientation particularly perceived fear of discrimination leading to social isolation after being diagnosed as HIV+ [24]. In our study, the social deprivation measure consists of indicators related to marital status and family structure particularly persons living alone, persons separated, divorced or widowed and single-parent families. However, considering lesbian, gay, bisexual and transgender (LGBT) forms of sociality and household composition, this social deprivation measure based on a hetero-sexual family structure may not reflect social deprivation among LGBT people. It is likely that applying this measure may lead to misclassification of LGBT people as being more socially deprived. Therefore, there is a need for development of social deprivation measures applicable to LGBT populations. Regarding material deprivation, it is possible that HIV+ monoinfected individuals are more educated and gainfully employed leading to a better financial status and making them more materially privileged. In our study, drug and alcohol use among HIV+ monoinfected individuals was slightly higher than the HIV/HCV negative group; however, HIV+monoinfected individuals had lower drug and alcohol use compared to the rest of the HIV/HCV positive groups, a finding contrary to some studies [25]. In this respect, HIV+ monoinfected individuals appear to represent a different risk group than HIV-/HCV+ seroconverters or coinfected individuals.

HIV-/HCV+ seroconverters and HIV+/HCV+ co-infected were materially and socially most deprived, had higher IDU, problematic alcohol use [13, 21, 26] and major mental illnesses. History of incarceration, homelessness and substance use has been reported as major predictors of HIV/HCV co-infection [27]. HIV-/HCV+ seroconverters constituted a group similar to the coinfected individuals in terms of risk factors. Both groups were younger in age and had similar odds of problematic alcohol use but the odds of IDU among HIV-/HCV+ seroconverters were higher. Our previous analyses have shown that OST and mental health counselling was associated with low HCV re-infection risk [28], which is higher in PWIDs and HIV coinfected individuals. Because of similarity in risk and ongoing vulnerabilities, HIV-/HCV+ seroconverters may be at higher risk of HIV infection, requiring intensive interventions.

The HIV-/HCV+ prevalent group showed a slightly different profile from HIV-/HCV+ seroconverters [17] and the rest of the testers. IDU was lower than HIV-/HCV+ seroconverters and co-infected individuals; however, problematic alcohol use had similar odds compared to HIV-/HCV+ seroconverters. Therefore, HIV-/HCV+ prevalent individuals could benefit from more alcohol related harm prevention services. This group was likely to be older and born between 1945 and 1964. Although HCV positivity rate is highest among the 1945–64 birth cohort, it has been declining over time suggesting a decreasing pool of undiagnosed prevalent HCV infections [17, 29].

In analyses stratified by time period, HIV-/HCV+ seroconverters, HIV-/HCV+ prevalent and coinfected individuals were strongly and consistently associated with IDU and drug use [17, 26] over time. This indicates that IDU has been a major risk factor for the transmission and sustainability of HCV or HIV infections over 20 years. Concomitantly, OST use has also increased in these groups. However, OST mainly started after HCV seroconversion in HIV-/HCV+ seroconverters representing a missed opportunity for prevention of infection. Once started, OST has shown effect on HCV re-infection and HIV infection prevention [16, 28, 30]. Problematic alcohol use was associated with almost all the HIV and HCV infected groups in the earlier years but less so after 2009. However, from 2005 onward, problematic alcohol use was associated only with HIV+ monoinfection. Although IDU was strongly associated with all the HCV + groups, there are various reasons for the decline in IDU particularly for the HIV-/HCV+ prevalent and coinfected individuals. This decline could be related to a shift to smoking that has happened over time as seen in other studies and surveys in British Columbia [31]. Particularly, in the case of crack cocaine use, an increase in crack cocaine smoking and decline in injecting drug use have been reported. In addition, as people age, their drug use pattern also changes. In our other analyses, we have seen that incidence of HCV was much lower in older birth cohorts compared to those born after 1975 [32]. Another phenomenon could be aging of the population who inject with fewer younger people initiating injecting. In British Columbia, especially in Vancouver city where the drug epidemic is concentrated, there has been an increase in OST, particularly among PWID and expansion of harm reduction programs in the city, as seen in an increase in OST uptake in our cohort (Fig 1). For declines in alcohol use, a recent study has shown a reduction in in both hazardous alcohol use and binge drinking after HCV treatment in a cohort receiving pegylated interferon (Peg-IFN)-based therapy [33]. Additionally, several studies have indicated that alcohol use declines with age [34, 35] and that there is excess mortality among heavy drinkers contributing to this age related decline [34]. It is possible that these could be the reasons for the declines in alcohol use observed in our cohort as well. However, this requires further investigation.

No association was found between mental illnesses or psychosis independently in our cohort for most testers. However, HIV-/HCV+ seroconverters were more likely to have a history of depression over time which is consistent with previous research [36]. HIV-/HCV+ seroconverters had history of IDU and problematic alcohol use; therefore, depression may be a function of these factors as well as HCV disease status. Further investigation is needed with more robust indicators of mental illness. In our cohort, HIV and HCV were mainly diseases of urban inner city as in other places [27, 37]. However, recent trends suggest some extension into rural areas as seen in recent surge in US. This trend was more evident for HIV+ monoinfected and HIV-/HCV+ seroconverters but less so for HCV+ prevalent which is mainly detected in baby boomers, many residing in rural areas. Some of this extension into rural areas could be related to improvement in services over time to detect infections. Active TB increased for HIV-/HCV+ seroconverters from 2009 onwards which may be a proxy for social disparities including homelessness and living in homeless shelters among HIV-/HCV+ seroconverters [38].

Our study provides valuable insights in the acquisition risk activities and the potential impact of social and material disparities. It documents the changing epidemiology of HCV and HIV infections over time (before and after 2000) and distribution of substance use and comorbidities within these infection groups. With the introduction of highly active antiretroviral therapy (HAART) in 1996, treatment improved drastically for HIV+ individuals. Prior to 1996, mortality among HIV+ individuals was high but after the introduction of HAART, HIV evolved into a chronic disease. For HCV, treatment was publically funded since the 2000s but with limited efficacy and uptake [39]. However, this will change with scale-up of HCV treatment with highly effective direct acting antivirals Our approach provides a unique population tool to see the impact of treatment, care and prevention programs on HIV and HCV infected populations.

Our study highlights the different evolution of syndemics for HIV, HCV (prevalent and seroconverters) and HIV/HCV, each of which will need targeted programs to support the combined and separate needs of each of the affected populations. In this regard, similar preventive strategies and services encompassing harm reduction, social support and psychiatric interventions could be targeted towards HIV-/HCV+ seroconverters and co-infected individuals, or those at risk for co-infection. These services could include access to stable, low barrier housing, income assistance, mental health and addictions services. For PWIDs, integrated models of care with screening and testing/treatment for HIV and HCV infections, management of psychiatric comorbidities and substance use including alcohol use could be initiated [16, 40]. Similarly, HIV-/HCV+ prevalent individuals could benefit from alcohol related harm prevention services to prevent its effect on liver disease progression. For HIV+ monoinfected individuals, prevention strategies and services should differ from those targeting persons with HCV with particular emphasis on access to care that is non-judgemental and free from stigma.

### Limitations

Results should be interpreted in the light of following methodological issues. The cohort did not include data on immigration status and Indigenous people, hence, we were not able to characterize infections and social conditions for these population groups who have been reported to have higher HBV and HCV infection rates. Similarly, those individuals who were not tested for HIV or HCV and may include some hard to reach populations are not included in this cohort. In this cohort, linkage rates were very high for HCV (>85%), especially in recent years. Linkage rates for those co-infected were also much higher than the overall HIV linkage rate; however, because of a low linkage rate for HIV especially before 2005, the HIV co-infection rate may have been underestimated. MSM status was not available for the entire cohort; hence, we could not use it for the current analysis. In our data which spanned more than 20 years, there were few HIV seroconverters and we were not able to run our regression models because of small sample size within the HCV+/HIV seroconverter group. Therefore, the HIV monoinfected group is a mix of incident and prevalent cases. Our HIV-/HCV- group included individuals tested negative for HCV but not tested for HIV and tested for HIV but not tested for HCV which may introduce some misclassification in our study.

In conclusion, we found co-occurrence and confluence of substance use, mental illnesses, co-infections and socioeconomic disparities which varied across HIV and HCV infection groups. Recognizing the variations in vulnerability between people monoinfected with HIV or HCV or coinfected with both can help inform necessary public health services. The most vulnerable groups were HIV-/HCV+seroconverters and HIV+/HCV+ co-infected individuals who were mainly PWIDs. Co-occurrence of infections, social conditions and syndemics require integration of testing, care, treatment and support services for those at risk for and with infections, substance use and mental illness.

## Supporting information

S1 Table Criteria and data sources for the BC Hepatitis Testers Cohort (BC-HTC). (DOCX)

S2 Table Definitions for comorbid conditions. (DOCX)

S3 Table Multivariate multinomial logistic regression model for factors associated with HIV and HCV infection status in the BC Hepatitis Testers Cohort (model excluding individuals not tested either for HIV or HCV) ^a^. (DOCX)

S4 Table Characteristics of participants by HIV and HCV status in BC Hepatitis Testers Cohort. (DOCX)

S5 Table Characteristics of testers by HCV and HIV test category in the British Columbia Hepatitis Testers Cohort stratified by year of diagnosis. (DOCX)

S6 Table Multivariate multinomial logistic regression model for factors associated with HIV and HCV infection status in the BC Hepatitis Testers Cohort presenting birth cohort instead of age ^a^. (DOCX)

S7 Table Multivariate multinomial logistic regression model for factors associated with HIV and HCV infection status in the BC Hepatitis Testers Cohort presenting depression and psychosis instead of mental illness ^a^. (DOCX)

S8 Table Multivariate multinomial logistic regression model for factors associated with HIV and HCV infection status in the BC Hepatitis Testers Cohort presenting assessments at the time of diagnosis ^a^. (DOCX)

S9 Table Multivariate multinomial logistic regression model for factors associated with HIV and HCV infection status in the BC Hepatitis Testers Cohort stratified by year of diagnosis ^a^. (DOCX)
