# Supplementary material for: A syndemic approach to assess the effect of substance use and social disparities on the evolution of HIV/HCV infections in British Columbia
Source: PLoS One. 2017 Aug 22;12(8):e0183609. doi: 10.1371/journal.pone.0183609 (PMC5568727; doi:10.1371/journal.pone.0183609)
Supplement: S1 Table — (DOCX) [file pone.0183609.s001.docx]

**S1 Table. Criteria and Data Sources for the BC Hepatitis Testers Cohort (BC-HTC)**

| **Criteria for Inclusion in BC-HTC** | |
| --- | --- |
| All individuals: | |
| - tested at the centralized provincial laboratory for HCV or HIV OR | |
| - reported by BC public health as a confirmed case of HCV OR | |
| - reported in BC enhanced surveillance system as a confirmed case of HIV or AIDS (all reports) OR | |
| - reported by BC public health as a confirmed case of HBV OR | |
| - included in BC Enhanced Strain Surveillance System (EHSSS) as an acute HBV or HCV case | |
| - All individuals meeting at least one the above criteria were linked internally across all their tests and case reports. Those with a valid personal health number (PHN) were then sent for deterministic linkage with province-wide Cancer and Ministry of Health (MoH) datasets | |
| **Provincial Communicable Disease Data Sources:** | **Data Date Ranges:** |
| BC-PHMRL HIV laboratory testing datasets (tests: ELISA, Western blot, NAAT, p24, culture) | 1988–2013 |
| BC-PHMRL HCV laboratory tests datasets (tests: antibody, HCV RNA, genotyping) | 1992–2013 |
| HIV/AIDS Information System (HAISYS) (public health HIV/AIDS case reports) | 1980–2013 |
| Integrated Public Health information System (iPHIS) (public health case reports of HCV, HBV, and TB) | 1990–2013 |
| Enhanced Strain Surveillance System (EHSSS) (risk factor data on a subset of acute HCV and acute HBV cases) | 2000–2013 |
| Cancer and MoH Administrative Data Sources: | Data Date Ranges: |
| BC Cancer Registry (BCCR) (primary tumour registry, excludes metastatic cancers) | 1970–2012 |
| Discharge Abstracts Dataset (DAD) (hospitalization records)^S1^ | 1985–2013Q1 |
| Medical Services Plan (MSP) (physician diagnostic and billing data)^S2^ | 1990–2012 |
| PharmaCare/PharmaNet (Pharma) (prescription drug dispensations)^S3, S4^ | 1985–2012 |
| BC Vital Statistics (VS) (deaths registry)^S5^ | 1985–2013 |
| The final BC-HTC comprises all individuals successfully linked on PHN to the MoH Client Roster^S6^ (a registry of all BC residents enrolled in the publicly-funded universal healthcare system) | |

HCV: Hepatitis C Virus; HBV: Hepatitis B Virus; HIV/AIDS: Human Immunodeficiency Virus/Acquired Immunodeficiency Syndrome; BC-PHMRL: BC Public Health Microbiology and Reference Laboratory: RNA: Ribonucleic Acid; PCR: Polymerase Chain Reaction.

**Supplementary References**:

1. British Columbia Ministry of Health [creator]. Discharge Abstract Database (Hospital Separations). British Columbia Ministry of Health [publisher]. Data Extract. MOH (2013). 2014. <http://www.health.gov.bc.ca/data/>
2. British Columbia Ministry of Health [creator]. Medical Services Plan (MSP) Payment Information File. British Columbia Ministry of Health [publisher]. Data Extract. MOH (2013). 2014. <http://www.health.gov.bc.ca/data/>
3. British Columbia Ministry of Health [creator]. PharmaCare. British Columbia Ministry of Health [publisher]. Data Extract. MOH (2013). 2014. <http://www.health.gov.bc.ca/data/>
4. British Columbia Ministry of Health [creator]. PharmaNet. British Columbia Ministry of Health [publisher]. Data Extract. MOH (2013). 2014. <http://www.health.gov.bc.ca/data/>
5. BC Vital Statistics Agency [creator]. Vital Statistics Deaths. BC Vital Statistics Agency [publisher]. Data Extract. BC Vital Statistics Agency (2014). 2014.
6. British Columbia Ministry of Health [creator]. Client Roster (Client Registry System/Enterprise Master Patient Index). British Columbia Ministry of Health [publisher]. Data Extract. MOH (2013). 2014. <http://www.health.gov.bc.ca/data/>
