# Supplementary material for: A syndemic approach to assess the effect of substance use and social disparities on the evolution of HIV/HCV infections in British Columbia
Source: PLoS One. 2017 Aug 22;12(8):e0183609. doi: 10.1371/journal.pone.0183609 (PMC5568727; doi:10.1371/journal.pone.0183609)
Supplement: S2 Table — (DOCX) [file pone.0183609.s002.docx]

**S2 Table. Definitions for comorbid conditions**

| **1. Major Mental Illness Diagnosis**  Major mental illness was flagged at the first occurrence of either 1 hospitalization diagnostic code OR 2 MSP diagnostic codes from a psychiatrist visit for schizophrenic, bipolar, delusional, nonorganic psychotic, adjustment, anxiety, dissociative, personality and major depressive disorders  Physician Billing Data: MSP ICD-9 diagnostic codes: starting with 295-298, 300-301, 308-309, 311 AND claim specialty = 3  Hospitalization Data: DAD1/ICD-9-CM: starting with 295-298, 300-301, 308-309, 311; DAD2/ICD-10-CA: starting with F20-F25, F28-F34, F38-F45, F48, F60-F61 |
| --- |
| **2. Problematic Alcohol Use**  Problematic alcohol use was defined at the first occurrence of 1 MSP or 1 hospitalization codes for major alcohol-related diagnoses including alcoholic mental disorders and dependence/abuse syndromes; alcoholic polyneuropathy, myopathy, cardiomyopathy; pseudo Cushing’s syndrome; or discharge to alcohol rehabilitation, counselling, or surveillance  Physician Billing Data: MSP ICD-9 diagnostic codes: starting with 291, 303, 3050, 3575, 4255  Hospitalization Data: DAD1/ICD-9-CM: starting with 291, 303, 3050,3575, 4255; DAD2/ICD-10-CA: starting with F10, E244, G312, G621, G721, I426, Z502, Z714 |
| **3. Illicit Drug Use**  Illicit Drug Use was defined at the first occurrence of 1 MSP or 1 hospitalization diagnostic codes for major drug-related diagnoses involving addiction, dependence, and drug-induced mental disorders; illicit drug use, or illicit use of prescribed drugs including: hallucinogens, barbiturates/tranquillizers, sedatives, hypnotics, anxiolytics, opioids, cocaine, amphetamine, volatile solvents; or discharge to drug rehabilitation, counselling, surveillance or methadone/buprenorphine substitution treatment  Physician Billing Data: MSP ICD-9 diagnostic codes: starting with 292, 304, 3053-3057 or exact codes V6542 or 39  Hospitalization Data: DAD1/ICD-9-CM: starting with 292, 304, 3053-3057 or exact code V6542; DAD2/ICD-10-CA: starting with F11-F16, F18-F19, Z715, Z503 |
