# Supplementary material for: A syndemic approach to assess the effect of substance use and social disparities on the evolution of HIV/HCV infections in British Columbia
Source: PLoS One. 2017 Aug 22;12(8):e0183609. doi: 10.1371/journal.pone.0183609 (PMC5568727; doi:10.1371/journal.pone.0183609)
Supplement: S3 Table — (DOCX) [file pone.0183609.s003.docx]

**S3 Table. Multivariate multinomial logistic regression model for factors associated with HIV and HCV infection status in the BC Hepatitis Testers Cohort (model excluding individuals not tested either for HIV or HCV) ^a^**

| **Variable** | **HIV+/HCV+** | **HIV+ / HCV-** | **HIV- / HCV+ prevalent** | **HIV- /HCV+ seroconverters** |
| --- | --- | --- | --- | --- |
|  | **OR (95% CI)** | **OR (95% CI)** | **OR (95% CI)** | **OR (95% CI)** |
| **Sex** |  |  |  |  |
| Female | 1 | 1 | 1 | 1 |
| Male | 2.8(2.65, 3.029) | 6.9(6.48, 7.47) | 2.2(2.12, 2.21) | 1.4 (1.32, 1.46) |
| **Age at diagnosis** |  |  |  |  |
| <15 | 0.7(0.40, 1.42) | 1.4(1.09, 1.70) | 0.3(0.28, 0.35) | 0.66(0.40, 1.10) |
| 15-24 | 4.3(3.48, 5.38) | 0.7(0.63, 0.81) | 0.2(0.18, 0.20) | 2.6(2.30-2.99) |
| 25-34 | 4.9(4.00, 6.04) | 1.3(1.17, 1.41) | 0.3(0.34, 0.36) | 2.45(2.17, 2.78) |
| 35-44 | 5.3(4.36, 6.58) | 1.6(1.44, 1.73) | 0.7(0.72, 0.76) | 2.1(1.84, 2.37) |
| 45-54 | 4.4(3.51, 5.42) | 1.7(1.53, 1.87) | 1.4(1.37, 1.46) | 1.8(1.57, 2.07) |
| >54 | 1 | 1 | 1 | 1 |
| **Urban** |  |  |  |  |
| No | 1 | 1 | 1 | 1 |
| Yes | 1.7(1.47, 1.89) | 1.8(1.61, 2.02) | 0.90(0.87, 0.93) | 0.99(0.91, 1.07) |
| **IDU^b^** |  |  |  |  |
| No | 1 | 1 | 1 | 1 |
| Yes | 10.8(10.04, 11.72) | 1.3(1.11, 1.43) | 5.6(5.38, 5.80) | 23.3(22.03, 24.73) |
| **Problematic alcohol use^b^** |  |  |  |  |
| No | 1 | 1 | 1 | 1 |
| Yes | 2.0(1.82, 2.23) | 0.9(0.75, 0.99) | 1.52(1.45, 1.58) | 1.8(1.66, 1.94) |
| **Major mental illness^b^** |  |  |  |  |
| No | 1 | 1 | 1 | 1 |
| Yes | 0.6(0.54, 0.67) | 1.1(0.97, 1.18) | 0.60(0.58, 0.63) | 0.8(0.79, 0.91) |
| **Active Tb^b^** |  |  |  |  |
| No | 1 | 1 | 1 | 1 |
| Yes | 1.3(0.62, 2.68) | 2.6(1.54, 4.31) | 0.7(0.49, 0.91) | 0.6(0.27, 1.46) |
| **Hepatitis B^b^** |  |  |  |  |
| No | 1 | 1 | 1 | 1 |
| Yes | 1.4(1.04, 1.90) | 1.8(1.38, 2.33) | 1.2(1.07, 1.39) | 2.8(2.21, 3.53) |
| **Year of diagnosis^b^** |  |  |  |  |
| >2009 | 1 | 1 | 1 | 1 |
| 2005-2009 | 6.2(5.12, 7.60) | 2.9(2.66, 3.130 | 3.3(3.23, 3.46) | 3.3(3.11, 3.58) |
| 2000-2004 | 28.1(23.4, 33.8) | 6.2(5.69, 6.69) | 9.4(9.10, 9.73) | 6.8(6.36, 7.34) |
| <2000 | 210.1(176.1, 250.7) | 20.8(19.3, 22.5) | 45.5(44.02, 46.99) | 8.3(7.66, 9.03) |
| **Social deprivation at time of test** |  |  |  |  |
| Q1 (most privileged) | 1 | 1 | 1 | 1 |
| Q2 | 1.5(1.28, 1.76) | 1.2(1.03, 1.31) | 1.1(1.07, 1.16) | 1.1(1.02, 1.29) |
| Q3 | 1.9(1.67, 2.25) | 1.2(1.11, 1.39) | 1.3(1.25, 1.35) | 1.6(1.40, 1.73) |
| Q4 | 2.6(2.29, 3.04) | 1.8(1.60, 1.98) | 1.4(1.34, 1.44) | 1.7(1.55, 1.89) |
| Q5 (most deprived) | 4.2(3.72, 4.85) | 3.0(2.75, 3.32) | 1.7(1.64, 1.76) | 2.6(2.38, 2.86) |
| **Material deprivation at time of test** |  |  |  |  |
| Q1 (most privileged) | 1 | 1 | 1 | 1 |
| Q2 | 1.1(0.94, 1.19) | 0.6(0.61, 0.71) | 1.4(1.31, 1.41) | 1.3(1.22, 1.48) |
| Q3 | 1.0(0.9, 1.14) | 0.5(0.49, 0.57) | 1.5(1.48, 1.58) | 1.4(1.28, 1.55) |
| Q4 | 1.4(1.23, 1.51) | 0.5(0.50, 0.58) | 1.8(1.72, 1.84) | 1.8(1.68, 2.0) |
| Q5 (most deprived) | 2.4(2.15, 2.59) | 0.68(0.63, 0.73) | 2.2(2.15, 2.30) | 2.3(2.17, 2.56) |

Abbreviations: IDU, injection drug use.

^a^ Reference group: HIV-/HCV-.

^b^ Factor assessed for past 3 years before diagnosis or last negative test.
