# Supplementary material for: A syndemic approach to assess the effect of substance use and social disparities on the evolution of HIV/HCV infections in British Columbia
Source: PLoS One. 2017 Aug 22;12(8):e0183609. doi: 10.1371/journal.pone.0183609 (PMC5568727; doi:10.1371/journal.pone.0183609)
Supplement: S4 Table — (DOCX) [file pone.0183609.s004.docx]

**S4 Table. Characteristics of participants by HIV and HCV status in BC Hepatitis Testers Cohort**

|  | **HIV+/HCV+** | **HIV+/HCV-** | **HIV-/HCV+ seroconverters** | **HIV-/HCV+ prevalent** | **HIV-/HCV-** |
| --- | --- | --- | --- | --- | --- |
| **Variable** | **N (%)** | **N (%)** | **N (%)** | **N (%)** | **N (%)** |
| **(row percent)** | 4639(0.3) | 6386(0.5) | 7013(0.5) | 56074(4.1) | 1302877(94.6) |
| **Sex** |  |  |  |  |  |
| Female | 1352(29.1) | 956(15) | 3120(44.5) | 19086(34) | 765346(58.7) |
| Male | 3286(70.8) | 5430(85) | 3893(55.5) | 36983(66) | 537363(41.2) |
| Unknown | 1(0) | 0(0) | 0(0) | 5(0) | 168(0) |
| **Birth year** |  |  |  |  |  |
| < 1945 | 106(2.3) | 474(7.4) | 123(1.8) | 6137(10.9) | 165710(12.7) |
| 1945-1964 | 2635(56.8) | 3058(47.9) | 1901(27.1) | 36762(65.6) | 356946(27.4) |
| > 1964 | 1898(40.9) | 2854(44.7) | 4989(71.1) | 13175(23.5) | 780221(59.9) |
| **Age at diagnosis** |  |  |  |  |  |
| <15 | 13(0.3) | 97(1.5) | 17(0.2) | 425(0.8) | 20573(1.6) |
| 15-24 | 580(12.5) | 460(7.2) | 1306(18.6) | 2298(4.1) | 174233(13.4) |
| 25-34 | 1702(36.7) | 1911(29.9) | 2686(38.3) | 9794(17.5) | 364607(28) |
| 35-44 | 1688(36.4) | 2089(32.7) | 1888(26.9) | 18076(32.2) | 280038(21.5) |
| 45-54 | 552(11.9) | 1178(18.4) | 810(11.5) | 15804(28.2) | 190520(14.6) |
| >54 | 104(2.2) | 651(10.2) | 306(4.4) | 9677(17.3) | 272906(20.9) |
| **Urban** |  |  |  |  |  |
| Unknown | 87(1.9) | 172(2.7) | 163(2.3) | 2531(4.5) | 29190(2.2) |
| No | 289(6.2) | 334(5.2) | 785(11.2) | 6590(11.8) | 131070(10.1) |
| Yes | 4263(91.9) | 5880(92.1) | 6065(86.5) | 46953(83.7) | 1142617(87.7) |
| **Social deprivation quintile at time of test** |  |  |  |  |  |
| Unknown | 150(3.2) | 202(3.2) | 178(2.5) | 2396(4.3) | 21030(1.6) |
| Q1 (most privileged) | 259(5.6) | 538(8.4) | 592(8.4) | 6111(10.9) | 235246(18.1) |
| Q2 | 405(8.7) | 656(10.3) | 675(9.6) | 7294(13) | 229929(17.6) |
| Q3 | 584(12.6) | 717(11.2) | 1024(14.6) | 9391(16.7) | 234744(18) |
| Q4 | 930(20) | 1205(18.9) | 1409(20.1) | 11626(20.7) | 269127(20.7) |
| Q5 (most deprived) | 2311(49.8) | 3068(48) | 3135(44.7) | 19256(34.3) | 312801(24) |
| **Material deprivation quintile at time of test** |  |  |  |  |  |
| Unknown | 150(3.2) | 202(3.2) | 178(2.5) | 2396(4.3) | 21030(1.6) |
| Q1 (most privileged) | 659(14.2) | 2101(32.9) | 826(11.8) | 7174(12.8) | 282648(21.7) |
| Q2 | 624(13.5) | 1075(16.8) | 970(13.8) | 8769(15.6) | 248346(19.1) |
| Q3 | 606(13.1) | 849(13.3) | 1112(15.9) | 10022(17.9) | 254554(19.5) |
| Q4 | 891(19.2) | 938(14.7) | 1665(23.7) | 12303(21.9) | 260362(20) |
| Q5 (most deprived) | 1709(36.8) | 1221(19.1) | 2262(32.3) | 15410(27.5) | 235937(18.1) |
| **Illicit Drug Use (baseline)** |  |  |  |  |  |
| No | 2382(51.3) | 5738(89.9) | 1895(27) | 39821(71) | 1214171(93.2) |
| Yes | 2257(48.7) | 648(10.1) | 5118(73) | 16253(29) | 88706(6.8) |
| **IDU (baseline)** |  |  |  |  |  |
| No | 3018(65.1) | 6021(94.3) | 2898(41.3) | 45737(81.6) | 1255056(96.3) |
| Yes | 1621(34.9) | 365(5.7) | 4115(58.7) | 10337(18.4) | 47821(3.7) |
| **OST (baseline)** |  |  |  |  |  |
| No | 4176(90.0) | 6348(99.4) | 5074(72.3) | 51957(92.7) | 1292022(99.2) |
| Yes | 463(9.9) | 38(0.6) | 1939(26.7) | 4117(7.3) | 10855(0.8) |
| **Major mental illness (baseline)** |  |  |  |  |  |
| No | 3817(82.3) | 5483(85.9) | 4488(64) | 48482(86.5) | 1141855(87.6) |
| Yes | 822(17.7) | 903(14.1) | 2525(36) | 7592(13.5) | 161022(12.4) |
| **Depression (baseline)** |  |  |  |  |  |
| No | 3108(67) | 4382(68.6) | 2718(38.8) | 37124(66.2) | 838455(64.4) |
| Yes | 1531(33) | 2004(31.4) | 4295(61.2) | 18950(33.8) | 464422(35.6) |
| **Psychosis (baseline)** |  |  |  |  |  |
| No | 4278(92.2) | 6148(96.3) | 6013(85.7) | 53371(95.2) | 1259521(96.7) |
| Yes | 361(7.8) | 238(3.7) | 1000(14.3) | 2703(4.8) | 43356(3.3) |
| **Problematic alcohol use (baseline)** |  |  |  |  |  |
| No | 3255(70.2) | 5828(91.3) | 4122(58.8) | 44633(79.6) | 1218702(93.5) |
| Yes | 1384(29.8) | 558(8.7) | 2891(41.2) | 11441(20.4) | 84175(6.5) |
| **Active TB (baseline)** |  |  |  |  |  |
| No | 4625(99.7) | 6364(99.7) | 7000(99.8) | 55975(99.8) | 1299599(99.7) |
| Yes | 14(0.3) | 22(0.3) | 13(0.2) | 99(0.2) | 3278(0.3) |
| **Hepatitis B (baseline)** |  |  |  |  |  |
| No | 4550(98.1) | 6277(98.3) | 6831(97.4) | 55452(98.9) | 1287388(98.8) |
| Yes | 89(1.9) | 109(1.7) | 182(2.6) | 622(1.1) | 15489(1.2) |

Abbreviations: IDU, injection drug use; OST, opioid substitution therapy.

Baseline: Factor assessed prior to diagnosis or last negative test.
