# Supplementary material for: A syndemic approach to assess the effect of substance use and social disparities on the evolution of HIV/HCV infections in British Columbia
Source: PLoS One. 2017 Aug 22;12(8):e0183609. doi: 10.1371/journal.pone.0183609 (PMC5568727; doi:10.1371/journal.pone.0183609)
Supplement: S5 Table — (DOCX) [file pone.0183609.s005.docx]

**S5 Table. Characteristics of testers by HCV and HIV test category in the British Columbia Hepatitis Testers Cohort stratified by year of diagnosis**

|  | **<2000** | **2000-2004** | **2005-2009** | **2010-2013** | **<2000** | **2000-2004** | **2005-2009** | **2010-2013** | **<2000** | **2000-2004** | **2005-2009** | **2010-2013** | **<2000** | **2000-2004** | **2005-2009** | **2010-2013** | **<2000** | **2000-2004** | **2005-2009** | **2010-2013** |
| --- | --- | --- | --- | --- | --- | --- | --- | --- | --- | --- | --- | --- | --- | --- | --- | --- | --- | --- | --- | --- |
| **Variable** | **HIV+/HCV+** | **HIV+/HCV+** | **HIV+/HCV+** | **HIV+/HCV+** | **HIV+/HCV-** | **HIV+/HCV-** | **HIV+/HCV-** | **HIV+/HCV-** | **HCV+ sero** | **HCV+ sero** | **HCV+ sero** | **HCV+ sero** | **HCV+ prevalent** | **HCV+ prevalent** | **HCV+ prevalent** | **HCV+ prevalent** | **HIV-/HCV-** | **HIV-/HCV-** | **HIV-/HCV-** | **HIV-/HCV-** |
|  | **N (%)** | **N (%)** | **N (%)** | **N (%)** | **N (%)** | **N (%)** | **N (%)** | **N (%)** | **N (%)** | **N (%)** | **N (%)** | **N (%)** | **N (%)** | **N (%)** | **N (%)** | **N (%)** | **N (%)** | **N (%)** | **N (%)** | **N (%)** |
| (Row percent) | 3137(2) | 940(0.5) | 423(0.1) | 139(0) | 2298(1.5) | 1585(0.8) | 1474(0.4) | 1029(0.2) | 1209(0.8) | 2153(1.1) | 2221(0.6) | 1430(0.2) | 26341(17) | 13568(7.1) | 10021(2.6) | 6144(0.9) | 122047(78.7) | 173972(90.5) | 367597(96.3) | 639261(98.7) |
| **Sex** |  |  |  |  |  |  |  |  |  |  |  |  |  |  |  |  |  |  |  |  |
| Female | 908(28.9) | 276(29.4) | 131(31) | 37(26.6) | 293(12.8) | 245(15.5) | 263(17.8) | 155(15.1) | 517(42.8) | 915(42.5) | 1017(45.8) | 671(46.9) | 9310(35.3) | 4478(33) | 3291(32.8) | 2007(32.7) | 66211(54.3) | 91177(52.4) | 216610(58.9) | 391348(61.2) |
| Male | 2229(71.1) | 664(70.6) | 292(69) | 101(72.7) | 2005(87.2) | 1340(84.5) | 1211(82.2) | 874(84.9) | 692(57.2) | 1238(57.5) | 1204(54.2) | 759(53.1) | 17027(64.6) | 9089(67) | 6730(67.2) | 4137(67.3) | 55748(45.7) | 82760(47.6) | 150969(41.1) | 247886(38.8) |
| Unknown | 0(0) | 0(0) | 0(0) | 1(0.7) | 0(0) | 0(0) | 0(0) | 0(0) | 0(0) | 0(0) | 0(0) | 0(0) | 4(0) | 1(0) | 0(0) | 0(0) | 88(0.1) | 35(0) | 18(0) | 27(0) |
| **Birth year** |  |  |  |  |  |  |  |  |  |  |  |  |  |  |  |  |  |  |  |  |
| < 1945 | 81(2.6) | 18(1.9) | 6(1.4) | 1(0.7) | 238(10.4) | 136(8.6) | 75(5.1) | 25(2.4) | 29(2.4) | 34(1.6) | 40(1.8) | 20(1.4) | 3551(13.5) | 1319(9.7) | 832(8.3) | 435(7.1) | 45844(37.6) | 32953(18.9) | 39369(10.7) | 47544(7.4) |
| 1945-1964 | 2026(64.6) | 436(46.4) | 135(31.9) | 38(27.3) | 1459(63.5) | 775(48.9) | 522(35.4) | 302(29.3) | 501(41.4) | 654(30.4) | 489(22) | 257(18) | 18016(68.4) | 8985(66.2) | 6144(61.3) | 3617(58.9) | 49066(40.2) | 69935(40.2) | 97212(26.4) | 140733(22) |
| > 1964 | 1030(32.8) | 486(51.7) | 282(66.7) | 100(71.9) | 601(26.2) | 674(42.5) | 877(59.5) | 702(68.2) | 679(56.2) | 1465(68) | 1692(76.2) | 1153(80.6) | 4774(18.1) | 3264(24.1) | 3045(30.4) | 2092(34) | 27137(22.2) | 71084(40.9) | 231016(62.8) | 450984(70.5) |
| **Age at diagnosis** |  |  |  |  |  |  |  |  |  |  |  |  |  |  |  |  |  |  |  |  |
| <15 | 11(0.4) | 2(0.2) | 0(0) | 0(0) | 46(2) | 17(1.1) | 24(1.6) | 10(1) | 8(0.7) | 4(0.2) | 2(0.1) | 3(0.2) | 217(0.8) | 94(0.7) | 84(0.8) | 30(0.5) | 4071(3.3) | 4345(2.5) | 5979(1.6) | 6178(1) |
| 15-24 | 398(12.7) | 124(13.2) | 51(12.1) | 7(5) | 157(6.8) | 93(5.9) | 116(7.9) | 94(9.1) | 254(21) | 491(22.8) | 361(16.3) | 200(14) | 1211(4.6) | 523(3.9) | 355(3.5) | 209(3.4) | 8370(6.9) | 18839(10.8) | 52007(14.1) | 95017(14.9) |
| 25-34 | 1255(40) | 292(31.1) | 105(24.8) | 50(36) | 816(35.5) | 409(25.8) | 389(26.4) | 297(28.9) | 528(43.7) | 796(37) | 858(38.6) | 504(35.2) | 6066(23) | 1948(14.4) | 1118(11.2) | 662(10.8) | 20348(16.7) | 36653(21.1) | 104535(28.4) | 203071(31.8) |
| 35-44 | 1136(36.2) | 347(36.9) | 167(39.5) | 38(27.3) | 789(34.3) | 575(36.3) | 467(31.7) | 258(25.1) | 301(24.9) | 588(27.3) | 621(28) | 378(26.4) | 10697(40.6) | 4208(31) | 2228(22.2) | 943(15.3) | 26803(22) | 42964(24.7) | 84019(22.9) | 126252(19.7) |
| 45-54 | 295(9.4) | 149(15.9) | 77(18.2) | 31(22.3) | 328(14.3) | 311(19.6) | 293(19.9) | 246(23.9) | 100(8.3) | 216(10) | 265(11.9) | 229(16) | 5299(20.1) | 4963(36.6) | 3740(37.3) | 1802(29.3) | 20221(16.6) | 32041(18.4) | 53093(14.4) | 85165(13.3) |
| >54 | 42(1.3) | 26(2.8) | 23(5.4) | 13(9.4) | 162(7) | 180(11.4) | 185(12.6) | 124(12.1) | 18(1.5) | 58(2.7) | 114(5.1) | 116(8.1) | 2851(10.8) | 1832(13.5) | 2496(24.9) | 2498(40.7) | 42234(34.6) | 39130(22.5) | 67964(18.5) | 123578(19.3) |
| **Urban** |  |  |  |  |  |  |  |  |  |  |  |  |  |  |  |  |  |  |  |  |
| Unknown | 55(1.8) | 13(1.4) | 11(2.6) | 8(5.8) | 75(3.3) | 16(1) | 34(2.3) | 47(4.6) | 25(2.1) | 48(2.2) | 48(2.2) | 41(2.9) | 838(3.2) | 572(4.2) | 537(5.4) | 578(9.4) | 2560(2.1) | 3049(1.8) | 6745(1.8) | 16661(2.6) |
| No | 162(5.2) | 70(7.4) | 45(10.6) | 12(8.6) | 102(4.4) | 78(4.9) | 83(5.6) | 71(6.9) | 116(9.6) | 237(11) | 276(12.4) | 156(10.9) | 2935(11.1) | 1593(11.7) | 1285(12.8) | 777(12.6) | 14348(11.8) | 17391(10) | 38660(10.5) | 60671(9.5) |
| Yes | 2920(93.1) | 857(91.2) | 367(86.8) | 119(85.6) | 2121(92.3) | 1491(94.1) | 1357(92.1) | 911(88.5) | 1068(88.3) | 1868(86.8) | 1897(85.4) | 1233(86.2) | 22568(85.7) | 11403(84) | 8199(81.8) | 4789(77.9) | 105139(86.1) | 153532(88.3) | 322192(87.6) | 561929(87.9) |
| **Social deprivation quintile** |  |  |  |  |  |  |  |  |  |  |  |  |  |  |  |  |  |  |  |  |
| Unknown | 106(3.4) | 27(2.9) | 9(2.1) | 8(5.8) | 89(3.9) | 25(1.6) | 35(2.4) | 53(5.2) | 41(3.4) | 58(2.7) | 44(2) | 35(2.4) | 806(3.1) | 592(4.4) | 456(4.6) | 542(8.8) | 1754(1.4) | 3216(1.8) | 3442(0.9) | 12618(2) |
| Q1 (most privileged) | 175(5.6) | 52(5.5) | 19(4.5) | 13(9.4) | 158(6.9) | 146(9.2) | 130(8.8) | 104(10.1) | 113(9.3) | 173(8) | 169(7.6) | 137(9.6) | 2551(9.7) | 1570(11.6) | 1183(11.8) | 807(13.1) | 19609(16.1) | 30924(17.8) | 66202(18) | 118511(18.5) |
| Q2 | 291(9.3) | 76(8.1) | 30(7.1) | 8(5.8) | 229(10) | 166(10.5) | 154(10.4) | 107(10.4) | 112(9.3) | 208(9.7) | 221(10) | 134(9.4) | 3456(13.1) | 1718(12.7) | 1331(13.3) | 789(12.8) | 22283(18.3) | 30742(17.7) | 64076(17.4) | 112828(17.6) |
| Q3 | 421(13.4) | 112(11.9) | 40(9.5) | 11(7.9) | 257(11.2) | 160(10.1) | 174(11.8) | 126(12.2) | 194(16) | 316(14.7) | 312(14) | 202(14.1) | 4604(17.5) | 2248(16.6) | 1606(16) | 933(15.2) | 23885(19.6) | 31964(18.4) | 66678(18.1) | 112217(17.6) |
| Q4 | 633(20.2) | 162(17.2) | 108(25.5) | 27(19.4) | 411(17.9) | 281(17.7) | 310(21) | 203(19.7) | 245(20.3) | 415(19.3) | 459(20.7) | 290(20.3) | 5693(21.6) | 2570(18.9) | 2121(21.2) | 1242(20.2) | 25279(20.7) | 33574(19.3) | 77061(21) | 133213(20.8) |
| Q5 (most deprived) | 1511(48.2) | 511(54.4) | 217(51.3) | 72(51.8) | 1154(50.2) | 807(50.9) | 671(45.5) | 436(42.4) | 504(41.7) | 983(45.7) | 1016(45.7) | 632(44.2) | 9231(35) | 4870(35.9) | 3324(33.2) | 1831(29.8) | 29237(24) | 43552(25) | 90138(24.5) | 149874(23.4) |
| **Material deprivation quintile** |  |  |  |  |  |  |  |  |  |  |  |  |  |  |  |  |  |  |  |  |
| Unknown | 106(3.4) | 27(2.9) | 9(2.1) | 8(5.8) | 89(3.9) | 25(1.6) | 35(2.4) | 53(5.2) | 41(3.4) | 58(2.7) | 44(2) | 35(2.4) | 806(3.1) | 592(4.4) | 456(4.6) | 542(8.8) | 1754(1.4) | 3216(1.8) | 3442(0.9) | 12618(2) |
| Q1 (most privileged) | 432(13.8) | 128(13.6) | 67(15.8) | 32(23) | 733(31.9) | 504(31.8) | 463(31.4) | 401(39) | 151(12.5) | 206(9.6) | 248(11.2) | 221(15.5) | 3398(12.9) | 1674(12.3) | 1282(12.8) | 820(13.3) | 23450(19.2) | 33783(19.4) | 75346(20.5) | 150069(23.5) |
| Q2 | 438(14) | 130(13.8) | 38(9) | 18(12.9) | 421(18.3) | 281(17.7) | 250(17) | 123(12) | 191(15.8) | 324(15) | 259(11.7) | 196(13.7) | 4397(16.7) | 1988(14.7) | 1416(14.1) | 968(15.8) | 23507(19.3) | 31944(18.4) | 68685(18.7) | 124210(19.4) |
| Q3 | 406(12.9) | 109(11.6) | 63(14.9) | 28(20.1) | 308(13.4) | 190(12) | 215(14.6) | 136(13.2) | 216(17.9) | 344(16) | 359(16.2) | 193(13.5) | 4710(17.9) | 2340(17.2) | 1906(19) | 1066(17.4) | 24361(20) | 33588(19.3) | 72035(19.6) | 124570(19.5) |
| Q4 | 537(17.1) | 229(24.4) | 107(25.3) | 18(12.9) | 296(12.9) | 262(16.5) | 225(15.3) | 155(15.1) | 263(21.8) | 534(24.8) | 588(26.5) | 280(19.6) | 5651(21.5) | 3103(22.9) | 2290(22.9) | 1259(20.5) | 24972(20.5) | 35557(20.4) | 74088(20.2) | 125745(19.7) |
| Q5 (most deprived) | 1218(38.8) | 317(33.7) | 139(32.9) | 35(25.2) | 451(19.6) | 323(20.4) | 286(19.4) | 161(15.6) | 347(28.7) | 687(31.9) | 723(32.6) | 505(35.3) | 7379(28) | 3871(28.5) | 2671(26.7) | 1489(24.2) | 24003(19.7) | 35884(20.6) | 74001(20.1) | 102049(16) |
| **Illicit Drug Use^a^** |  |  |  |  |  |  |  |  |  |  |  |  |  |  |  |  |  |  |  |  |
| No | 2032(64.8) | 622(66.2) | 283(66.9) | 107(77) | 2228(97) | 1502(94.8) | 1370(92.9) | 970(94.3) | 598(49.5) | 983(45.7) | 1000(45) | 691(48.3) | 20917(79.4) | 11600(85.5) | 8885(88.7) | 5613(91.4) | 120223(98.5) | 170141(97.8) | 359330(97.8) | 622561(97.4) |
| Yes | 1105(35.2) | 318(33.8) | 140(33.1) | 32(23) | 70(3) | 83(5.2) | 104(7.1) | 59(5.7) | 611(50.5) | 1170(54.3) | 1221(55) | 739(51.7) | 5424(20.6) | 1968(14.5) | 1136(11.3) | 531(8.6) | 1824(1.5) | 3831(2.2) | 8267(2.2) | 16700(2.6) |
| **IDU^a^** |  |  |  |  |  |  |  |  |  |  |  |  |  |  |  |  |  |  |  |  |
| No | 2217(70.7) | 653(69.5) | 271(64.1) | 101(72.7) | 2239(97.4) | 1518(95.8) | 1379(93.6) | 973(94.6) | 735(60.8) | 1132(52.6) | 1052(47.4) | 645(45.1) | 22109(83.9) | 11844(87.3) | 8890(88.7) | 5537(90.1) | 120955(99.1) | 171232(98.4) | 360663(98.1) | 623256(97.5) |
| Yes | 920(29.3) | 287(30.5) | 152(35.9) | 38(27.3) | 59(2.6) | 67(4.2) | 95(6.4) | 56(5.4) | 474(39.2) | 1021(47.4) | 1169(52.6) | 785(54.9) | 4232(16.1) | 1724(12.7) | 1131(11.3) | 607(9.9) | 1092(0.9) | 2740(1.6) | 6934(1.9) | 16005(2.5) |
| **OST^a^** |  |  |  |  |  |  |  |  |  |  |  |  |  |  |  |  |  |  |  |  |
| No | 2913(92.9) | 833(88.6) | 372(87.9) | 120(86.3) | 2295(99.9) | 1575(99.4) | 1464(99.3) | 1021(99.2) | 1076(89.0) | 1655(76.9) | 1649(74.3) | 873(61.1) | 24816(94.2) | 12744(93.9) | 9554(95.3) | 5783(94.1) | 121923(99.9) | 173449(99.7) | 366349(99.7) | 632528(98.9) |
| Yes | 224(7.1) | 107(11.4) | 51(12.1) | 19(13.7) | 3(0.1) | 10(0.6) | 10(0.7) | 8(0.8) | 133(11.0) | 498(23.1) | 572(25.7) | 557(38.9) | 1525(5.8) | 824(6.1) | 467(4.7) | 361(5.9) | 124(0.1) | 523(0.3) | 1248(0.3) | 6733(1.1) |
| **Major Mental Illness^a^** |  |  |  |  |  |  |  |  |  |  |  |  |  |  |  |  |  |  |  |  |
| No | 2739(87.3) | 835(88.8) | 371(87.7) | 125(89.9) | 2091(91) | 1451(91.5) | 1367(92.7) | 957(93) | 958(79.2) | 1646(76.5) | 1805(81.3) | 1103(77.1) | 23892(90.7) | 12538(92.4) | 9384(93.6) | 5755(93.7) | 115345(94.5) | 163200(93.8) | 347435(94.5) | 602574(94.3) |
| Yes | 398(12.7) | 105(11.2) | 52(12.3) | 14(10.1) | 207(9) | 134(8.5) | 107(7.3) | 72(7) | 251(20.8) | 507(23.5) | 416(18.7) | 327(22.9) | 2449(9.3) | 1030(7.6) | 637(6.4) | 389(6.3) | 6702(5.5) | 10772(6.2) | 20162(5.5) | 36687(5.7) |
| **Depression^a^** |  |  |  |  |  |  |  |  |  |  |  |  |  |  |  |  |  |  |  |  |
| No | 2395(76.3) | 732(77.9) | 339(80.1) | 124(89.2) | 1851(80.5) | 1287(81.2) | 1264(85.8) | 881(85.6) | 729(60.3) | 1219(56.6) | 1433(64.5) | 881(61.6) | 20088(76.3) | 10948(80.7) | 8434(84.2) | 5324(86.7) | 101667(83.3) | 141813(81.5) | 306196(83.3) | 542316(84.8) |
| Yes | 742(23.7) | 208(22.1) | 84(19.9) | 15(10.8) | 447(19.5) | 298(18.8) | 210(14.2) | 148(14.4) | 480(39.7) | 934(43.4) | 788(35.5) | 549(38.4) | 6253(23.7) | 2620(19.3) | 1587(15.8) | 820(13.3) | 20380(16.7) | 32159(18.5) | 61401(16.7) | 96945(15.2) |
| **Psychosis^a^** |  |  |  |  |  |  |  |  |  |  |  |  |  |  |  |  |  |  |  |  |
| No | 3005(95.8) | 907(96.5) | 401(94.8) | 134(96.4) | 2258(98.3) | 1555(98.1) | 1441(97.8) | 1010(98.2) | 1154(95.5) | 2018(93.7) | 2084(93.8) | 1309(91.5) | 25765(97.8) | 13227(97.5) | 9806(97.9) | 6020(98) | 120660(98.9) | 171256(98.4) | 362748(98.7) | 630616(98.6) |
| Yes | 132(4.2) | 33(3.5) | 22(5.2) | 5(3.6) | 40(1.7) | 30(1.9) | 33(2.2) | 19(1.8) | 55(4.5) | 135(6.3) | 137(6.2) | 121(8.5) | 576(2.2) | 341(2.5) | 215(2.1) | 124(2) | 1387(1.1) | 2716(1.6) | 4849(1.3) | 8645(1.4) |
| **Problematic alcohol use^a^** |  |  |  |  |  |  |  |  |  |  |  |  |  |  |  |  |  |  |  |  |
| No | 2631(83.9) | 818(87) | 382(90.3) | 131(94.2) | 2223(96.7) | 1531(96.6) | 1406(95.4) | 988(96) | 965(79.8) | 1793(83.3) | 1864(83.9) | 1219(85.2) | 23499(89.2) | 12677(93.4) | 9392(93.7) | 5813(94.6) | 119141(97.6) | 169823(97.6) | 360485(98.1) | 627591(98.2) |
| Yes | 506(16.1) | 122(13) | 41(9.7) | 8(5.8) | 75(3.3) | 54(3.4) | 68(4.6) | 41(4) | 244(20.2) | 360(16.7) | 357(16.1) | 211(14.8) | 2842(10.8) | 891(6.6) | 629(6.3) | 331(5.4) | 2906(2.4) | 4149(2.4) | 7112(1.9) | 11670(1.8) |
| **Active Tb^a^** |  |  |  |  |  |  |  |  |  |  |  |  |  |  |  |  |  |  |  |  |
| No | 3128(99.7) | 939(99.9) | 423(100) | 139(100) | 2291(99.7) | 1582(99.8) | 1470(99.7) | 1027(99.8) | 1209(100) | 2151(99.9) | 2220(100) | 1427(99.8) | 26310(99.9) | 13556(99.9) | 10010(99.9) | 6140(99.9) | 121765(99.8) | 173478(99.7) | 367007(99.8) | 638528(99.9) |
| Yes | 9(0.3) | 1(0.1) | 0(0) | 0(0) | 7(0.3) | 3(0.2) | 4(0.3) | 2(0.2) | 0(0) | 2(0.1) | 1(0) | 3(0.2) | 31(0.1) | 12(0.1) | 11(0.1) | 4(0.1) | 282(0.2) | 494(0.3) | 590(0.2) | 733(0.1) |
| **Hepatitis B^a^** |  |  |  |  |  |  |  |  |  |  |  |  |  |  |  |  |  |  |  |  |
| No | 3107(99) | 926(98.5) | 415(98.1) | 139(100) | 2261(98.4) | 1570(99.1) | 1461(99.1) | 1027(99.8) | 1162(96.1) | 2118(98.4) | 2212(99.6) | 1429(99.9) | 26082(99) | 13465(99.2) | 9989(99.7) | 6137(99.9) | 121011(99.2) | 172142(98.9) | 366066(99.6) | 637469(99.7) |
| Yes | 30(1) | 14(1.5) | 8(1.9) | 0(0) | 37(1.6) | 15(0.9) | 13(0.9) | 2(0.2) | 47(3.9) | 35(1.6) | 9(0.4) | 1(0.1) | 259(1) | 103(0.8) | 32(0.3) | 7(0.1) | 1036(0.8) | 1830(1.1) | 1531(0.4) | 1792(0.3) |

Abbreviations: IDU, injection drug use; OST, opioid substitution therapy.

^a^ Factor assessed for past 3 years before diagnosis or last negative test.
