# Supplementary material for: A syndemic approach to assess the effect of substance use and social disparities on the evolution of HIV/HCV infections in British Columbia
Source: PLoS One. 2017 Aug 22;12(8):e0183609. doi: 10.1371/journal.pone.0183609 (PMC5568727; doi:10.1371/journal.pone.0183609)
Supplement: S6 Table — (DOCX) [file pone.0183609.s006.docx]

**S6 Table. Multivariate multinomial logistic regression model for factors associated with HIV and HCV infection status in the BC Hepatitis Testers Cohort presenting birth cohort instead of age ^a^**

| **Variable** | **HIV+/HCV+** | **HIV+ / HCV-** | **HIV- /HCV+ prevalent** | **HIV- / HCV+ seroconverters** |
| --- | --- | --- | --- | --- |
|  | **OR (95% CI)** | **OR (95% CI)** | **OR (95% CI)** | **OR (95% CI)** |
| **Sex** |  |  |  |  |
| Female | 1 | 1 | 1 | 1 |
| Male | 2.8(2.62 , 2.99) | 7.2(6.71 , 7.72) | 2.3(2.22 , 2.31) | 1.4(1.33 , 1.47) |
| **Birth year** |  |  |  |  |
| > 1964 | 1 | 1 | 1 | 1 |
| 1945-1964 | 1.2(1.14 , 1.3) | 1.3(1.24 , 1.38) | 3.5(3.39 , 3.55) | 0.6(0.6 , 0.67) |
| < 1945 | 0.1(0.06 , 0.08) | 0.3(0.28 , 0.34) | 0.9(0.88 , 0.94) | 0.1(0.08 , 0.12) |
| **Urban** |  |  |  |  |
| No | 1 | 1 | 1 | 1 |
| Yes | 1.7(1.52 , 1.94) | 1.8(1.64 , 2.05) | 0.9(0.89 , 0.94) | 1.0(0.93 , 1.1) |
| **IDU^b^** |  |  |  |  |
| No | 1 | 1 | 1 | 1 |
| Yes | 17.1(15.89 , 18.56) | 1.8(1.57 , 2.04) | 8.1(7.81 , 8.39) | 34.2(32.2 , 36.2) |
| **Problematic alcohol use^b^** |  |  |  |  |
| No | 1 | 1 | 1 | 1 |
| Yes | 2.3(2.09 , 2.54) | 1(0.89 , 1.17) | 1.8(1.71 , 1.9) | 1.9(1.79 , 2.09) |
| **Major mental illness^b^** |  |  |  |  |
| No | 1 | 1 | 1 | 1 |
| Yes | 0.7(0.59 , 0.73) | 1.2(1.11 , 1.35) | 0.7(0.66 , 0.72) | 0.9(0.82 , 0.95) |
| **Active TB^b^** |  |  |  |  |
| No | 1 | 1 | 1 | 1 |
| Yes | 0.6(0.31 , 1.26) | 1.4(0.84 , 2.3) | 0.4(0.31 , 0.55) | 0.4(0.18 , 0.97) |
| **Hepatitis B^b^** |  |  |  |  |
| No | 1 | 1 | 1 | 1 |
| Yes | 1(0.73 , 1.30) | 1.2(0.96 , 1.59) | 0.8(0.7 , 0.88) | 2(1.62 , 2.55) |
| **Year of diagnosis** |  |  |  |  |
| >2009 | 1 | 1 | 1 | 1 |
| 2000-2004 | 27.9(23.23 , 33.61) | 5.4(5.02 , 5.91) | 6.6(6.39 , 6.83) | 7.6(7.08 , 8.18) |
| 2005-2009 | 5.8(4.72 , 7.01) | 2.6(2.36 , 2.78) | 2.7(2.63 , 2.81) | 3.1(2.93 , 3.37) |
| <2000 | 160.1(134.1 , 191.2) | 13.1(12.12 , 14.19) | 18.9(18.36 , 19.56) | 7.7(7.13 , 8.44) |
| **Social deprivation at time of test** |  |  |  |  |
| Q1 (most privileged) | 1 | 1 | 1 | 1 |
| Q2 | 1.6(1.34 , 1.84) | 1.2(1.09 , 1.38) | 1.2(1.12 , 1.21) | 1.2(1.07 , 1.34) |
| Q3 | 2.1(1.80 , 2.43) | 1.4(1.21 , 1.52) | 1.4(1.34 , 1.44) | 1.7(1.49 , 1.83) |
| Q4 | 3.0(2.63 , 3.48) | 2.1(1.86 , 2.29) | 1.6(1.52 , 1.63) | 1.9(1.7 , 2.07) |
| Q5 (most deprived) | 5.3(4.61 , 5.99) | 3.8(3.48 , 4.19) | 2.1(2.02 , 2.15) | 3.0(2.75 , 3.30) |
| **Material deprivation quintile at time of test** |  |  |  |  |
| Q1 (most privileged) | 1 | 1 | 1 | 1 |
| Q2 | 1(0.93 , 1.17) | 0.6(0.59 , 0.69) | 1.3(1.27 , 1.36) | 1.3(1.2 , 1.45) |
| Q3 | 1(0.88 , 1.11) | 0.5(0.46 , 0.55) | 1.4(1.4 , 1.5) | 1.4(1.25 , 1.50) |
| Q4 | 1.3(1.19 , 1.46) | 0.5(0.47 , 0.55) | 1.7(1.61 , 1.72) | 1.8(1.63 , 1.93) |
| Q5 (most deprived) | 2.3(2.09 , 2.52) | 0.6(0.6 , 0.7) | 2.1(2.02 , 2.15) | 2.3(2.12 , 2.50) |

Abbreviations: IDU, injection drug use

^a^ Reference group: HIV-/HCV-.

^b^ Factor assessed for past 3 years before diagnosis or last negative test.
