# Supplementary material for: A syndemic approach to assess the effect of substance use and social disparities on the evolution of HIV/HCV infections in British Columbia
Source: PLoS One. 2017 Aug 22;12(8):e0183609. doi: 10.1371/journal.pone.0183609 (PMC5568727; doi:10.1371/journal.pone.0183609)
Supplement: S7 Table — (DOCX) [file pone.0183609.s007.docx]

**S7 Table. Multivariate multinomial logistic regression model for factors associated with HIV and HCV infection status in the BC Hepatitis Testers Cohort presenting depression and psychosis instead of mental illness ^a^**

| **Variable** | **HIV+/HCV+** | **HIV+ / HCV-** | **HIV- / HCV+ prevalent** | **HIV- /HCV+ seroconverters** |
| --- | --- | --- | --- | --- |
|  | **OR (95% CI)** | **OR (95% CI)** | **OR (95% CI)** | **OR (95% CI)** |
| **Sex** |  |  |  |  |
| Female | 1 | 1 | 1 | 1 |
| Male | 2.8(2.64 , 3.02) | 7.6(7.06 , 8.13) | 2.3(2.26 , 2.35) | 1.5(1.45 , 1.60) |
| **Age at diagnosis** |  |  |  |  |
| >54 | 1 | 1 | 1 | 1 |
| <15 | 1.1(0.57 , 2.00) | 1.6(1.27 , 1.97) | 0.4(0.38 , 0.47) | 0.7(0.44 , 1.2) |
| 15-24 | 13.8(11.1 , 17.2) | 1.7(1.47 , 1.89) | 0.5(0.49 , 0.54) | 5.3(4.64 , 6.03) |
| 25-34 | 16.8(13.73 , 20.71) | 3.3(2.98 , 3.58) | 1.03(1.00 , 1.07) | 5.2(4.63 , 5.92) |
| 35-44 | 16.0(13.08 , 19.72) | 3.6(3.33 ,3.99) | 2(1.88 , 1.99) | 4.2(3.66 , 4.70) |
| 45-54 | 8.9(7.16 , 11.05) | 2.9(2.63 , 3.2) | 2.6(2.49 , 2.64) | 2.8(2.43 , 3.19) |
| **Urban** |  |  |  |  |
| No | 1 | 1 | 1 | 1 |
| Yes | 1.7(1.48 , 1.90) | 1.78(1.6 , 2) | 0.9(0.88 , 0.93) | 1(0.94 , 1.09) |
| **IDU^b^** |  |  |  |  |
| No | 1 | 1 | 1 | 1 |
| Yes | 14.3(13.26 , 15.48) | 1.7(1.51 , 1.95) | 7.4(7.19 , 7.71) | 29.7(28.07 , 31.53) |
| **Problematic alcohol use^b^** |  |  |  |  |
| No | 1 | 1 | 1 | 1 |
| Yes | 2.3(2.05 , 2.5) | 1.1(0.92 , 1.21) | 1.7(1.66 , 1.80) | 1.8(1.66 , 1.94) |
| **Depression^b^** |  |  |  |  |
| No | 1 | 1 | 1 | 1 |
| Yes | 0.8(0.75 , 0.88) | 1.2(1.1 , 1.26) | 0.94(0.91 , 0.96) | 1.4(1.33 , 1.49) |
| **Psychosis^b^** |  |  |  |  |
| No | 1 | 1 | 1 | 1 |
| Yes | 0.8(0.69 , 0.96) | 0.9(0.71 , 1.04) | 0.7(0.66 , 0.76) | 0.7(0.64 , 0.8) |
| **Active TB^b^** |  |  |  |  |
| No | 1 | 1 | 1 | 1 |
| Yes | 0.6(0.29 , 1.20) | 1.37(0.83 , 2.26) | 0.4(0.3 , 0.53) | 0.4(0.19 , 1) |
| **Hepatitis B^b^** |  |  |  |  |
| No | 1 | 1 | 1 | 1 |
| Yes | 0.9(0.7 , 1.26) | 1.2(0.96 , 1.59) | 0.8(0.74 , 0.93) | 2.07(1.65 , 2.59) |
| Year of diagnosis |  |  |  |  |
| >2009 | 1 | 1 | 1 | 1 |
| 2005-2009 | 5.6(4.6 , 6.84) | 2.5(2.29 , 2.7) | 2.8(2.74 , 2.94) | 3(2.83 , 3.25) |
| 2000-2004 | 26.6(22.12 , 31.98) | 5.2(4.76 , 5.60) | 7.6(7.35 , 7.85) | 6.5(6.08 , 7.01) |
| <2000 | 138.7(116.3 , 165.5) | 11.6(10.8 , 12.56) | 22.6(21.98 , 23.38) | 5.6(5.14 , 6.05) |
| **Social deprivation at time**  **of test** |  |  |  |  |
| Q1 (most privileged) | 1 | 1 | 1 | 1 |
| Q2 | 1.6(1.33 , 1.83) | 1.2(1.09 , 1.37) | 1.2(1.12 , 1.21) | 1.2(1.06 , 1.33) |
| Q3 | 2.1(1.78 , 2.40) | 1.3(1.19 , 1.5) | 1.4(1.34 , 1.44) | 1.6(1.46 , 1.80) |
| Q4 | 3(2.56 , 3.40) | 2(1.81 , 2.23) | 1.6(1.51 , 1.62) | 1.9(1.68 , 2.05) |
| Q5 (most deprived) | 5.1(4.5 , 5.80) | 3.7(3.34 , 4.03) | 2.1(2.00 , 2.13) | 3(2.72 , 3.26) |
| **Material deprivation quintile**  **at time of test** |  |  |  |  |
| Q1 (most privileged) | 1 | 1 | 1 | 1 |
| Q2 | 1.1(0.94 , 1.18) | 0.7(0.6 , 0.7) | 1.3(1.3 , 1.39) | 1.3(1.2 , 1.46) |
| Q3 | 1(0.89 , 1.11) | 0.5(0.47 , 0.56) | 1.5(1.42 , 1.52) | 1.4(1.25 , 1.50) |
| Q4 | 1.3(1.2 , 1.47) | 0.5(0.48 , 0.56) | 1.7(1.64 , 1.74) | 1.8(1.63 , 1.94) |
| Q5 (most deprived) | 2.3(2.09 , 2.52) | 0.7(0.62 , 0.71) | 2.1(2.07 , 2.20) | 2.3(2.15 , 2.54) |

Abbreviations: IDU, injection drug use

^a^ Reference group: HIV-/HCV-.

^b^ Factor assessed for past 3 years before diagnosis or last negative test.
