# Supplementary material for: A syndemic approach to assess the effect of substance use and social disparities on the evolution of HIV/HCV infections in British Columbia
Source: PLoS One. 2017 Aug 22;12(8):e0183609. doi: 10.1371/journal.pone.0183609 (PMC5568727; doi:10.1371/journal.pone.0183609)
Supplement: S8 Table — (DOCX) [file pone.0183609.s008.docx]

**S8 Table. Multivariate multinomial logistic regression model for factors associated with HIV and HCV infection status in the BC Hepatitis Testers Cohort presenting assessments at the time of diagnosis ^a^**

| **Variable** | **HIV+/HCV+** | **HIV+ / HCV-** | **HIV- / HCV+ prevalent** | **HIV- /HCV+ seroconverters** |
| --- | --- | --- | --- | --- |
|  | **OR (95% CI)** | **OR (95% CI)** | **OR (95% CI)** | **OR (95% CI)** |
| **Sex** |  |  |  |  |
| Female | 1 | 1 | 1 | 1 |
| Male | 2.8(2.58 , 2.95) | 7.5(7.01 , 8.08) | 2.2(2.18 , 2.27) | 1.4(1.36 , 1.5) |
| **Age at diagnosis** |  |  |  |  |
| <15 | 1.2(0.64 , 2.25) | 1.6(1.27 , 1.97) | 0.4(0.4 , 0.5) | 0.9(0.56 , 1.53) |
| 15-24 | 15.1(12.11 , 18.70) | 1.7(1.49 , 1.91) | 0.5(0.52 , 0.57) | 6.3(5.55 , 7.20) |
| 25-34 | 16.8(13.67 , 20.62) | 3.3(3.01 , 3.62) | 1(1.01 , 1.07) | 5.5(4.88 , 6.25) |
| 35-44 | 15.3(12.42 , 18.72) | 3.7(3.36 , 4.03) | 1.9(1.84 , 1.94) | 4.1(3.59 , 4.61) |
| 45-54 | 8.4(6.73 , 10.38) | 2.9(2.63 , 3.20) | 2.5(2.42 , 2.56) | 2.6(2.27 , 2.98) |
| >54 | 1 | 1 | 1 | 1 |
| **Urban** |  |  |  |  |
| No | 1 | 1 | 1 | 1 |
| Yes | 1.8(1.55 , 1.99) | 1.8(1.59 , 1.99) | 0.9(0.90 , 0.96) | 1.1(0.99 , 1.16) |
| **IDU^b^** |  |  |  |  |
| No | 1 | 1 | 1 | 1 |
| Yes | 10.6(9.85 , 11.45) | 1.3(1.20 , 1.53) | 5.9(5.76 , 6.13) | 22.7(21.33 , 24.09) |
| **Problematic alcohol use^b^** |  |  |  |  |
| No | 1 | 1 | 1 | 1 |
| Yes | 2.8(2.55 , 2.97) | 1(0.89 , 1.07) | 2.1(2.06 , 2.17) | 2.8(2.59 , 2.92) |
| **Major mental illness^b^** |  |  |  |  |
| No | 1 | 1 | 1 | 1 |
| Yes | 0.7(0.60 , 0.72) | 1.3(1.18 , 1.37) | 0.7(0.64 , 0.68) | 1(0.93 , 1.05) |
| **Active TB^b^** |  |  |  |  |
| No | 1 | 1 | 1 | 1 |
| Yes | 0.8(0.46 , 1.46) | 1.4(0.93 , 2.19) | 0.5(0.42 , 0.65) | 0.7(0.38 , 1.19) |
| **Hepatitis B^b^** |  |  |  |  |
| No | 1 | 1 | 1 | 1 |
| Yes | 1.3(1.04 , 1.64) | 1.3(1.04 , 1.54) | 0.8(0.75 , 0.90) | 2.4(2.06 , 2.84) |
| **Year of diagnosis** |  |  |  |  |
| >2009 | 1 | 1 | 1 | 1 |
| 2005-2009 | 6(4.89 , 7.25) | 2.5(2.31 , 2.72) | 3(2.86 , 3.07) | 3.3(3.1 , 3.56) |
| 2000-2004 | 29.1(24.19 , 34.98) | 5.2(4.82 , 5.67) | 8.1(7.82 , 8.36) | 7.7(7.12 , 8.22) |
| <2000 | 157.9(132.3 , 188.41) | 11.9(10.99 , 12.83) | 24.6(23.85 , 25.39) | 7.1(6.6 , 7.72) |
| **Social deprivation at time of test** |  |  |  |  |
| Q1 (most privileged) | 1 | 1 | 1 | 1 |
| Q2 | 1.5(1.31 , 1.8) | 1.2(1.09 , 1.37) | 1.2(1.11 , 1.2) | 1.2(1.03 , 1.3) |
| Q3 | 2(1.75 , 2.36) | 1.3(1.19 , 1.5) | 1.4(1.32 , 1.42) | 1.6(1.4 , 1.73) |
| Q4 | 2.9(2.48 , 3.29) | 2(1.81 , 2.23) | 1.5(1.47 , 1.58) | 1.8(1.59 , 1.94) |
| Q5 (most deprived) | 4.8(4.21 , 5.5) | 3.7(3.33 , 4.01) | 2(1.92 , 2.04) | 2.7(2.48 , 2.97) |
| **Material deprivation quintile at time of test** |  |  |  |  |
| Q1 (most privileged) | 1 | 1 | 1 | 1 |
| Q2 | 1(0.91 , 1.15) | 0.7(0.6 , 0.7) | 1.3(1.27 , 1.36) | 1.3(1.16 , 1.41) |
| Q3 | 0.9(0.85 , 1.06) | 0.5(0.47 , 0.56) | 1.4(1.38 , 1.48) | 1.3(1.19 , 1.43) |
| Q4 | 1.3(1.13 , 1.4) | 0.5(0.48 , 0.57) | 1.6(1.59 , 1.69) | 1.7(1.54 , 1.84) |
| Q5 (most deprived) | 2.1(1.95 , 2.36) | 0.7(0.62 , 0.71) | 2(1.96 , 2.08) | 2.1(1.95 , 2.3) |

Abbreviations: IDU, injection drug use.

^a^ Reference group: HIV-/HCV-.

^b^ Factor assessed before diagnosis or last negative test.
