# Supplementary material for: A syndemic approach to assess the effect of substance use and social disparities on the evolution of HIV/HCV infections in British Columbia
Source: PLoS One. 2017 Aug 22;12(8):e0183609. doi: 10.1371/journal.pone.0183609 (PMC5568727; doi:10.1371/journal.pone.0183609)
Supplement: S9 Table — (DOCX) [file pone.0183609.s009.docx]

**S9 Table. Multivariate multinomial logistic regression model for factors associated with HIV and HCV infection status in the BC Hepatitis Testers Cohort stratified by year of diagnosis ^a^**

|  | <2000 | 2000-2004 | 2005-2009 | 2010-2013 | <2000 | 2000-2004 | 2005-2009 | 2010-2013 | <2000 | 2000-2004 | 2005-2009 | 2010-2013 | <2000 | 2000-2004 | 2005-2009 | 2010-2013 |
| --- | --- | --- | --- | --- | --- | --- | --- | --- | --- | --- | --- | --- | --- | --- | --- | --- |
| **Variable** | **HIV+/HCV+** | **HIV+/HCV+** | **HIV+/HCV+** | **HIV+/HCV+** | **HIV+ / HCV-** | **HIV+ / HCV-** | **HIV+ / HCV-** | **HIV+ / HCV-** | **HIV- / HCV+ prevalent** | **HIV- / HCV+ prevalent** | **HIV- / HCV+ prevalent** | **HIV- / HCV+ prevalent** | **HIV- /HCV+ seroconverters** | **HIV- /HCV+ seroconverters** | **HIV- /HCV+ seroconverters** | **HIV- /HCV+ seroconverters** |
|  | **OR (95% CI)** | **OR (95% CI)** | **OR (95% CI)** | **OR (95% CI)** | **OR (95% CI)** | **OR (95% CI)** | **OR (95% CI)** | **OR (95% CI)** | **OR (95% CI)** | **OR (95% CI)** | **OR (95% CI)** | **OR (95% CI)** | **OR (95% CI)** | **OR (95% CI)** | **OR (95% CI)** | **OR (95% CI)** |
| **Sex** |  |  |  |  |  |  |  |  |  |  |  |  |  |  |  |  |
| Female | 1 | 1 | 1 | 1 | 1 | 1 | 1 | 1 | 1 | 1 | 1 | 1 | 1 | 1 | 1 | 1 |
| Male | 3.1(2.88 , 3.41) | 2.4(2.09 , 2.80) | 2.7(2.17 , 3.33) | 3.7(2.45 , 5.46) | 8.9(7.85 , 10.14) | 6.3(5.46 , 7.21) | 6.2(5.44 , 7.15) | 9(7.52 , 10.78) | 2.3(2.26 , 2.4) | 2.1(2.04 , 2.20) | 2.3(2.18 , 2.39) | 2.4(2.23 , 2.5) | 1.9(1.70 , 2.18) | 1.5(1.37 , 1.65) | 1.4(1.25 , 1.51) | 1.3(1.14 , 1.42) |
| **Birth year** |  |  |  |  |  |  |  |  |  |  |  |  |  |  |  |  |
| > 1964 | 1 | 1 | 1 | 1 | 1 | 1 | 1 | 1 | 1 | 1 | 1 | 1 | 1 | 1 | 1 | 1 |
| 1945-1964 | 2.7(2.43 , 3.12) | 1.5(1.11 , 1.92) | 1.3(0.87 , 1.82) | 1.7(0.66 , 4.51) | 2.7(2.36 , 3.16) | 1(0.8 , 1.16) | 1.3(1.04 , 1.59) | 1.5(1.11 , 2.16) | 2.3(2.14 , 2.44) | 1.8(1.67 , 2) | 2(1.85 , 2.23) | 2.2(1.86 , 2.49) | 0.7(0.54 , 0.85) | 0.9(0.72 , 1.06) | 1.3(1.09 , 1.61) | 0.9(0.66 , 1.21) |
| < 1945 | 1.9(1.31 , 2.79) | 0.7(0.26 , 1.69) | 0.4(0.13 , 0.97) | 0.3(0.03 , 2.50) | 3.7(2.7 , 5.02) | 0.6(0.38 , 0.83) | 0.6(0.44 , 0.92) | 0.6(0.37 , 1.12) | 1.6(1.47 , 1.85) | 0.9(0.78 , 1.03) | 0.8(0.67 , 0.86) | 0.7(0.61 , 0.88) | 0.4(0.22 , 0.84) | 0.3(0.15 , 0.48) | 0.7(0.45 , 1.09) | 0.4(0.25 , 0.77) |
| **Urban** |  |  |  |  |  |  |  |  |  |  |  |  |  |  |  |  |
| No | 1 | 1 | 1 | 1 | 1 | 1 | 1 | 1 | 1 | 1 | 1 | 1 | 1 | 1 | 1 | 1 |
| Yes | 2.0(1.71 , 2.37) | 1.6(1.20 , 2.02) | 1.1(0.8 , 1.51) | 1(0.57 , 1.9) | 2.2(1.81 , 2.72) | 1.8(1.46 , 2.32) | 1.8(1.45 , 2.28) | 1.2(0.93 , 1.51) | 1.0(0.97 , 1.06) | 0.9(0.83 , 0.94) | 0.8(0.79 , 0.9) | 0.7(0.67 , 0.78) | 1.2(0.94 , 1.40) | 1(0.9 , 1.18) | 1(0.89 , 1.16) | 1(0.81 , 1.15) |
| **IDU^b^** |  |  |  |  |  |  |  |  |  |  |  |  |  |  |  |  |
| No | 1 | 1 | 1 | 1 | 1 | 1 | 1 | 1 | 1 | 1 | 1 | 1 | 1 | 1 | 1 | 1 |
| Yes | 23.6(21.14 , 26.34) | 17.7(15.06 , 20.98) | 20.4(16.15 , 25.76) | 13.4(8.75 , 20.69) | 1.9(1.42 , 2.50) | 2.2(1.66 , 2.79) | 2.5(1.99 , 3.13) | 1.4(1.09 , 1.97) | 13.8(12.8 , 14.9) | 9.1(8.44 , 9.78) | 6.4(5.91 , 7.0) | 4.3(3.91 , 4.79) | 28.3(24.4 , 32.8) | 28.2(25.3 , 31.4) | 39.2(35.3 , 43.4) | 32(28.5 , 36.9) |
| **Problematic alcohol use^b^** |  |  |  |  |  |  |  |  |  |  |  |  |  |  |  |  |
| No | 1 | 1 | 1 | 1 | 1 | 1 | 1 | 1 | 1 | 1 | 1 | 1 | 1 | 1 | 1 | 1 |
| Yes | 3.3(2.92 , 3.73) | 2.3(1.9 , 2.92) | 1.2(0.84 , 1.72) | 1(0.44 , 2.00) | 0.9(0.7 , 1.14) | 1(0.73 , 1.29) | 1.4(1.10 , 1.86) | 1.4(0.99 , 1.92) | 2.5(2.34 , 2.67) | 1.4(1.25 , 1.49) | 1.3(1.22 , 1.47) | 1.4(1.21 , 1.56) | 3.6(3.07 , 4.33) | 2.0(1.72 , 2.32) | 1.5(1.35 , 1.8) | 1.2(0.98 , 1.36) |
| **Depression^b^** |  |  |  |  |  |  |  |  |  |  |  |  |  |  |  |  |
| No | 1 | 1 | 1 | 1 | 1 | 1 | 1 | 1 | 1 | 1 | 1 | 1 | 1 | 1 | 1 | 1 |
| Yes | 1(0.92 , 1.12) | 0.7(0.61 , 0.87) | 0.6(0.48 , 0.81) | 0.4(0.20 , 0.64) | 1.6(1.42 , 1.77) | 1.2(1.01 , 1.32) | 0.8(0.72 , 0.99) | 1(0.86 , 1.24) | 1.2(1.15 , 1.24) | 0.8(0.79 , 0.87) | 0.7(0.68 , 0.77) | 0.7(0.66 , 0.78) | 2.1(1.81 , 2.37) | 1.7(1.56 , 1.93) | 1.1(1 , 1.22) | 1.2(1.03 , 1.32) |
| **Psychosis^b^** |  |  |  |  |  |  |  |  |  |  |  |  |  |  |  |  |
| No | 1 | 1 | 1 | 1 | 1 | 1 | 1 | 1 | 1 | 1 | 1 | 1 | 1 | 1 | 1 | 1 |
| Yes | 1.1(0.92 , 1.44) | 0.5(0.37 , 0.78) | 0.8(0.47 , 1.20) | 0.7(0.27 , 1.8) | 1.1(0.78 , 1.53) | 0.7(0.51 , 1.08) | 0.9(0.62 , 1.28) | 0.7(0.47 , 1.2) | 0.9(0.78 , 1.0) | 0.7(0.65 , 0.85) | 0.7(0.6 , 0.81) | 0.7(0.60 , 0.87) | 1(0.74 , 1.37) | 0.6(0.5 , 0.75) | 0.6(0.51 , 0.75) | 0.9(0.74 , 1.11) |
| **Active TB^b^** |  |  |  |  |  |  |  |  |  |  |  |  |  |  |  |  |
| No | 1 | 1 | 1 | 1 | 1 | 1 | 1 | 1 | 1 | 1 | 1 | 1 | 1 | 1 | 1 | 1 |
| Yes | 0.6(0.26 , 1.24) | 0.3(0.04 , 2.38) | 0(0 , > 10) | 0(0 , > 10) | 1.2(0.55 , 2.58) | 0.9(0.29 , 2.84) | 1.9(0.7 , 5.06) | 2.2(0.54 , 8.7) | 0.3(0.23 , 0.53) | 0.3(0.18 , 0.59) | 0.5(0.28 , 0.94) | 0.4(0.16 , 1.12) | 0(0 , > 10) | 0.39(0.09 , 1.65) | 0.3(0.04 , 2.07) | 2.7(0.82 , 8.7) |
| **Hepatitis B^b^** |  |  |  |  |  |  |  |  |  |  |  |  |  |  |  |  |
| No | 1 | 1 | 1 | 1 | 1 | 1 | 1 | 1 | 1 | 1 | 1 | 1 | 1 | 1 | 1 | 1 |
| Yes | 0.7(0.45 , 0.97) | 1.5(0.85 , 2.5) | 4.2(2.07 , 8.69) | 0(0 , > 10) | 1.3(0.94 , 1.91) | 0.8(0.49 , 1.42) | 1.8(1.04 , 3.14) | 0.6(0.16 , 2.5) | 0.9(0.74 , 1.0) | 0.7(0.54 , 0.83) | 0.6(0.45 , 0.91) | 0.4(0.18 , 0.81) | 3.1(2.22 , 4.29) | 1.9(1.28 , 2.69) | 1.1(0.57 , 2.23) | 0.5(0.06 , 3.25) |
| **Age at diagnosis** |  |  |  |  |  |  |  |  |  |  |  |  |  |  |  |  |
| <15 | 4.8(2.14 , 10.98) | 0.6(0.13 , 3.09) | 0(0 , > 10) | 0(0 , > 10) | 10.8(6.79 , 17.04) | 0.5(0.28 , 0.93) | 1.5(0.94 , 2.54) | 2.2(1.03 , 4.75) | 1.3(1.10 , 1.60) | 0.5(0.36 , 0.59) | 0.4(0.34 , 0.57) | 0.3(0.22 , 0.52) | 2.5(0.87 , 7.49) | 0.3(0.11 , 0.93) | 0.3(0.06 , 1.07) | 0.4(0.09 , 1.52) |
| 15-24 | 72.9(44.17 , 120.28) | 6.5(2.89 , 14.71) | 1.8(0.92 , 3.46) | 1(0.26 , 3.82) | 16.1(10.90 , 23.71) | 0.7(0.44 , 1.0) | 0.9(0.62 , 1.23) | 1.6(1.04 , 2.54) | 3(2.54 , 3.35) | 0.5(0.43 , 0.59) | 0.2(0.19 , 0.26) | 0.2(0.14 , 0.22) | 20.8(9.12 , 47.73) | 4.2(2.61 , 6.6) | 2.8(2.0 , 3.83) | 1.2(0.8 , 1.74) |
| 25-34 | 61.3(37.9 , 99.37) | 7.8(3.50 , 17.39) | 1.9(1.01 , 3.6) | 2.9(0.91 , 9.4) | 24.4(17.49 , 34.10) | 1.4(0.98 , 2.06) | 1.5(1.13 , 2.07) | 2.5(1.64 , 3.7) | 4.5(3.98 , 5.0) | 1(0.86 , 1.13) | 0.4(0.33 , 0.42) | 0.3(0.22 , 0.31) | 18(7.96 , 40.24) | 3.5(2.20 , 5.57) | 3.3(2.41 , 4.56) | 1.3(0.92 , 1.96) |
| 35-44 | 25.5(15.87 , 41.24) | 6.5(3.07 , 13.89) | 3.4(1.89 , 5.96) | 3.3(1.0 , 10.54) | 11(7.96 , 15.23) | 1.8(1.34 , 2.54) | 2(1.53 , 2.61) | 2.9(1.91 , 4.34) | 4(3.51 , 4.34) | 1.2(1.11 , 1.36) | 0.8(0.7 , 0.84) | 0.6(0.5 , 0.71) | 11.2(5.08 , 24.86) | 2.7(1.76 , 4.15) | 2.8(2.1 , 3.76) | 1.5(1.05 , 2.24) |
| 45-54 | 9.7(6.19 , 15.39) | 3.9(1.82 , 8.33) | 2(1.19 , 3.43) | 2.4(1.2 , 4.95) | 5.6(4.2 , 7.49) | 1.4(1 , 1.91) | 1.4(1.16 , 1.81) | 2.5(1.92 , 3.14) | 2.8(2.54 , 3.08) | 1.8(1.65 , 2.02) | 1.1(1.07 , 1.22) | 0.8(0.78 , 0.9) | 5.9(2.78 , 12.5) | 1.6(1.04 , 2.49) | 1.6(1.19 , 2.04) | 1.5(1.14 , 1.93) |
| >54 | 1 | 1 | 1 | 1 | 1 | 1 | 1 | 1 | 1 | 1 | 1 | 1 | 1 | 1 | 1 | 1 |
| **Social deprivation quintile at time of test** |  |  |  |  |  |  |  |  |  |  |  |  |  |  |  |  |
| Q1 (most privileged) | 1 | 1 | 1 | 1 | 1 | 1 | 1 | 1 | 1 | 1 | 1 | 1 | 1 | 1 | 1 | 1 |
| Q2 | 1.6(1.31 , 1.93) | 1.6(1.11 , 2.24) | 1.7(0.94 , 2.97) | 0.7(0.29 , 1.69) | 1.3(1.08 , 1.63) | 1.2(0.93 , 1.46) | 1.3(1.01 , 1.63) | 1.1(0.81 , 1.4) | 1.2(1.13 , 1.27) | 1.1(1.07 , 1.23) | 1.2(1.08 , 1.27) | 1.1(0.99 , 1.21) | 0.9(0.71 , 1.22) | 1.2(0.99 , 1.51) | 1.3(1.08 , 1.63) | 1.2(0.94 , 1.53) |
| Q3 | 2.1(1.75 , 2.52) | 2.2(1.56 , 3.04) | 2.2(1.26 , 3.76) | 1(0.44 , 2.2) | 1.5(1.19 , 1.79) | 1.1(0.91 , 1.43) | 1.5(1.16 , 1.85) | 1.3(1 , 1.7) | 1.4(1.36 , 1.53) | 1.4(1.30 , 1.5) | 1.3(1.23 , 1.44) | 1.3(1.21 , 1.47) | 1.4(1.12 , 1.83) | 1.6(1.32 , 1.95) | 1.7(1.42 , 2.1) | 1.7(1.36 , 2.14) |
| Q4 | 3(2.45 , 3.47) | 2.9(2.14 , 4.04) | 4.5(2.74 , 7.31) | 1.9(0.99 , 3.79) | 2.1(1.77 , 2.58) | 1.9(1.52 , 2.29) | 2.2(1.81 , 2.75) | 1.8(1.38 , 2.23) | 1.7(1.6 , 1.76) | 1.5(1.42 , 1.63) | 1.5(1.37 , 1.59) | 1.4(1.28 , 1.54) | 1.6(1.24 , 1.99) | 1.9(1.58 , 2.3) | 1.9(1.58 , 2.28) | 1.9(1.55 , 2.36) |
| Q5 (most deprived) | 4.8(4.09 , 5.68) | 6.1(4.6 , 8.14) | 6.9(4.29 , 11.02) | 4(2.2 , 7.27) | 4.3(3.63 , 5.10) | 3.6(3.04 , 4.35) | 3.7(3.02 , 4.43) | 2.7(2.21 , 3.41) | 2.2(2.05 , 2.3) | 2.1(2.01 , 2.28) | 2(1.82 , 2.09) | 1.7(1.61 , 1.9) | 2.5(1.99 , 3.06) | 3.0(2.55 , 3.58) | 3.1(2.64 , 3.70) | 2.9(2.43 , 3.56) |
| **Material deprivation quintile at time of test** |  |  |  |  |  |  |  |  |  |  |  |  |  |  |  |  |
| Q1 (most privileged) | 1 | 1 | 1 | 1 | 1 | 1 | 1 | 1 | 1 | 1 | 1 | 1 | 1 | 1 | 1 | 1 |
| Q2 | 1.1(0.94 , 1.24) | 1.2(0.92 , 1.52) | 0.7(0.46 , 1.02) | 1(0.54 , 1.74) | 0.7(0.57 , 0.74) | 0.7(0.59 , 0.8) | 0.7(0.58 , 0.8) | 0.5(0.39 , 0.58) | 1.3(1.24 , 1.38) | 1.3(1.21 , 1.4) | 1.2(1.14 , 1.33) | 1.5(1.37 , 1.66) | 1.3(1.03 , 1.6) | 1.7(1.40 , 2.03) | 1.2(0.97 , 1.39) | 1.2(0.97 , 1.45) |
| Q3 | 0.9(0.82 , 1.09) | 1(0.76 , 1.27) | 1(0.73 , 1.47) | 1.4(0.85 , 2.4) | 0.5(0.41 , 0.54) | 0.5(0.41 , 0.57) | 0.6(0.48 , 0.67) | 0.5(0.42 , 0.63) | 1.3(1.27 , 1.41) | 1.5(1.38 , 1.57) | 1.5(1.44 , 1.66) | 1.6(1.44 , 1.73) | 1.36(1.08 , 1.69) | 1.7(1.36 , 2) | 1.4(1.16 , 1.63) | 1.1(0.87 , 1.29) |
| Q4 | 1.2(1.02 , 1.34) | 1.7(1.36 , 2.11) | 1.5(1.13 , 2.09) | 0.8(0.46 , 1.47) | 0.4(0.37 , 0.49) | 0.6(0.49 , 0.67) | 0.5(0.46 , 0.63) | 0.6(0.48 , 0.69) | 1.5(1.44 , 1.6) | 1.7(1.63 , 1.85) | 1.8(1.64 , 1.89) | 1.9(1.69 , 2.03) | 1.5(1.21 , 1.83) | 2.1(1.78 , 2.51) | 2(1.7 , 2.31) | 1.4(1.16 , 1.66) |
| Q5 (most deprived) | 2.4(2.11 , 2.67) | 2.2(1.82 , 2.76) | 2(1.46 , 2.65) | 1.5(0.92 , 2.47) | 0.6(0.52 , 0.66) | 0.7(0.57 , 0.76) | 0.7(0.62 , 0.84) | 0.7(0.56 , 0.81) | 2(1.85 , 2.04) | 2.2(2.03 , 2.29) | 2(1.9 , 2.18) | 2.5(2.30 , 2.75) | 1.8(1.50 , 2.23) | 2.7(2.27 , 3.15) | 2.4(2.03 , 2.73) | 2.3(1.98 , 2.74) |

Abbreviations: IDU, injection drug use

^a^ Reference group: HIV-/HCV-.

^b^ Factor assessed for past 3 years before diagnosis or last negative test.
